# Amine-Catalyzed Decarboxylative Aldol Reaction of β-Ketocarboxylic Acids with Trifluoropyruvates

**DOI:** 10.3390/molecules24152773

**Published:** 2019-07-30

**Authors:** Ryouta Kawanishi, Shinya Hattori, Seiji Iwasa, Kazutaka Shibatomi

**Affiliations:** Department of Applied Chemistry and Life Science, Toyohashi University of Technology, 1-1 Hibarigaoka, Tempaku-cho, Toyohashi 441-8580, Japan

**Keywords:** decarboxylation, aldol reaction, trifluoromethyl compounds, cinchona alkaloids, enantioselective synthesis, chiral amine catalyst

## Abstract

Decarboxylative aldol reaction of aliphatic carboxylic acids is a useful method for C–C bond formation because carboxylic acids are an easily available class of compounds. In this study, we found that the decarboxylative aldol reaction of tertiary β-ketocarboxylic acids and trifluoropyruvates proceeded smoothly to yield the corresponding aldol products in high yields and with high diastereoselectivity in the presence of a tertiary amine catalyst. In this reaction, we efficiently constructed a quaternary carbon center and an adjacent trifluoromethylated carbon center. This protocol was also extended to an enantioselective reaction with a chiral amine catalyst, and the desired product was obtained with up to 73% enantioselectivity.

## 1. Introduction

**Figure 1:**
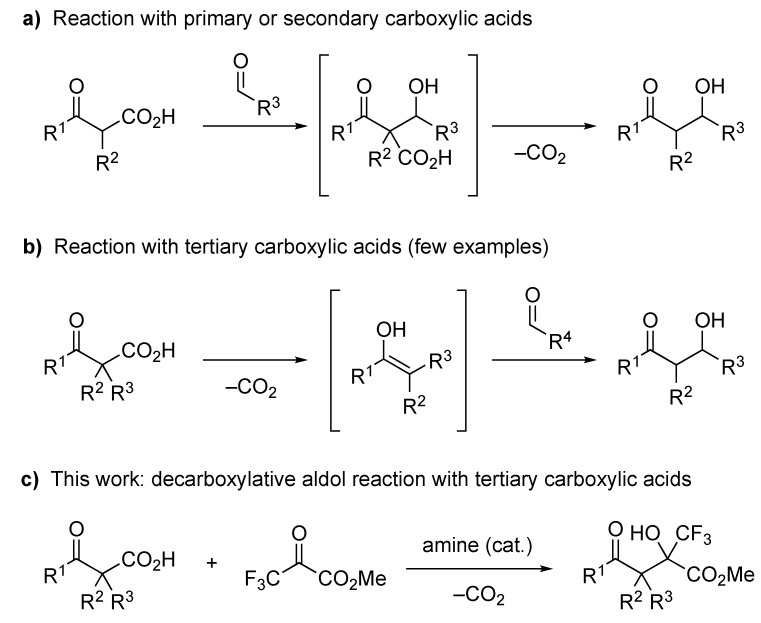
Decarboxylative aldol reactions of β-oxocarboxylic acids: (**a**) Reaction with primary and secondary carboxylic acids; (**b**) Reaction with tertiary carboxylic acids; (**c**) This work.

Decarboxylative functionalization of aliphatic carboxylic acids is a highly useful method in synthetic organic chemistry [1,2,3,4,5,6,7,8,9] because the carboxyl group is a fundamental and easily available functional group. In particular, the decarboxylative aldol reaction of β-oxocarboxylic acids has been intensively studied in recent years [2,5,6,10,11] because these acids easily release carbon dioxide to serve as a formal enolate equivalent [12,13,14,15,16,17,18,19,20]. A number of decarboxylative aldol reactions with primary and secondary β-oxocarboxylic acids have been reported, including a catalytic enantioselective version [2,5,6,10,11,21,22,23]. Most of these reactions are thought to proceed via the aldol adduct of the β-oxocarboxylic acid and the acceptor, followed by decarboxylative protonation, to yield the desired product (Figure 1a). On the other hand, decarboxylative aldol reactions with tertiary carboxylic acids have rarely been reported. To the best of our knowledge, there is only one report on the decarboxylative aldol reaction with tertiary β-oxocarboxylic acids to form an all-carbon quaternary carbon center [24], and there is no report on the enantioselective version, although there are some examples of reactions with α,α-difluoro- or α-monofluorocarboxylic acids to form a fluorinated tetrasubstituted carbon center [25,26,27,28]. These reactions proceed via an enol (or enolate) intermediate formed by decarboxylation of the β-oxocarboxylic acid, and the intermediate reacts with the aldol acceptor to give the desired product (Figure 1b). Therefore, the difficulty in the decarboxylative aldol reaction of tertiary carboxylic acids probably arises from the high steric hinderance of the trisubstituted enolate intermediate. Recently, our research group found that the decarboxylative halogenation of β-ketocarboxylic acids and 2-pyridylcarboxylic acids proceeds even with tertiary carboxylic acids as the substrate [29,30,31,32]. We hypothesized that this smooth decarboxylative functionalization of tertiary carboxylic acids was due to the high electrophilicity of the electrophilic halogenating agents, such as *N*-chlorosuccinimide (NCS), *N*-fluorobenzenesulfonimide (NFSI), and Selectfluor. Our previous results encouraged us to develop a decarboxylative aldol reaction of β-ketocarboxylic acids by employing a highly electrophilic aldol acceptor. Trifluoropyruvates are known to be good aldol acceptors with high electrophilicity [33,34,35,36,37,38], and the resulting aldol products, which have a trifluoromethylated stereogenic carbon center, are attractive building blocks for medicinally relevant compounds [39,40,41,42,43,44]. Therefore, we examined the decarboxylative aldol reaction of tertiary β-ketocarboxylic acids with trifluoropyruvates to form the corresponding aldol adduct (Figure 1c), and we disclose the results herein, including the application of this reaction to enantioselective synthesis.

## 2. Results and Discussion

First, we chose tetralone-derived β-ketocarboxylic acids 1a as the model substrate and then screened various amine catalysts for the decarboxylative aldol reaction with methyl trifluoropyruvate as the acceptor (Table 1). Tertiary amines showed superior catalytic activity than primary and secondary amines (entries 1–8). Bicyclic tertiary amines with bridgehead nitrogen(s) showed particularly high reactivity, diastereoselectivity (92:8–93:7 dr), and chemoselectivity between aldol product 2a and protonated product 3a (entries 7 and 8). Taking the reactivity, selectivity, and cost into account, we chose 1,4-diazabicyclo [2.2.2]octane (DABCO) as the best catalyst. Decreasing the catalyst loading to 15 mol% led to a satisfactory level of reactivity and selectivity (entry 9). Optimization of various reaction solvents revealed that toluene was the best choice for the model reaction (entries 9–13).

**Figure 2:**
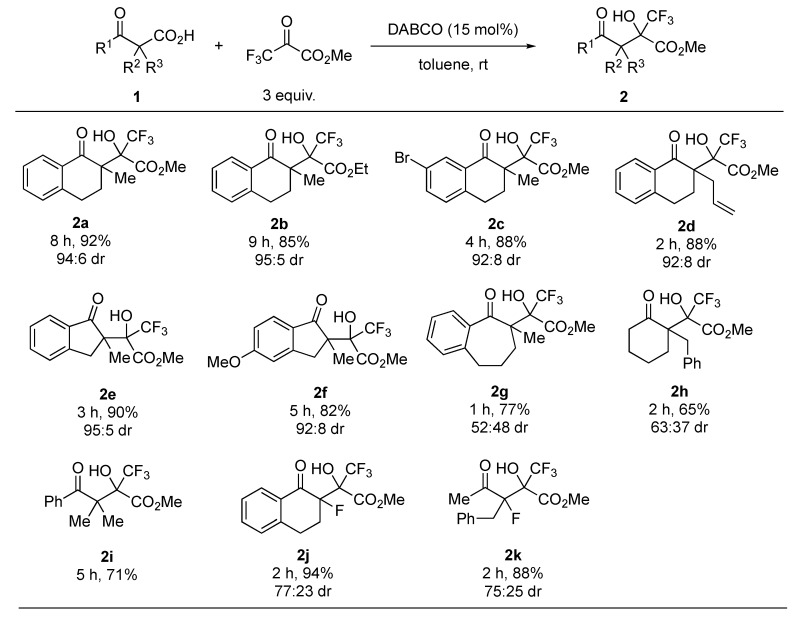
Substrate scope ^1^. ^1^ All reactions were carried out at room temperature in toluene with 3 equiv. of trifluoropyruvates in the presence of 15 mol% DABCO. Isolated yields were described. Diastereomeric ratios were determined by ^1^H-NMR analyses.

With the optimized reaction conditions in hand, next we focused on the substrate scope of the reaction. The results are summarized in Figure 2. The use of ethyl trifluoropyruvate gave the corresponding aldol product 2b in good yield and with high diastereoselectivity. Substituents on the benzoyl moiety or at the α-position of the carbonyl group had little effect on the reactivity and diastereoselectivity (2c and 2d). Indanone-derived β-ketocarboxylic acids 1e and 1f also afforded the corresponding products 2e and 2f in high yields and with high diastereoselectivity, whereas benzosuberone- and cyclohexanone-derived substrates 1g and 1h yielded poor diastereoselectivity of the respective products 2g and 2h. This reaction was also applicable to an acyclic substrate 1i. In addition, the reaction of α-fluoro-β-ketocarboxylic acids furnished the desired tertiary fluorides 2j and 2k in high yields, and with good diastereoselectivity.

Next, we attempted to apply our method to catalytic enantioselective synthesis. First, we employed chiral primary amine C1 for the reaction of 1a with methyl trifluoropyruvate in toluene, because C1 showed high enantioselectivity in the decarboxylative chlorination of 1a in our previous study. To our surprise, the present reaction only yielded the desired aldol adduct in a moderate yield, with no enantioselectivity (Table 2, entry 1). The probable reason for this result was that the primary amine moiety of C1 reacted with the highly reactive keto carbonyl group of methyl trifluoropyruvate to form an imine or a hemiaminal, thereby causing C1 to lose its catalytic activity. Considering that quinuclidine and DABCO showed high catalytic activity (Table 1), we used cinchona alkaloids and their derivatives in the reaction of 1a and methyl trifluoropyruvate. As expected, all the tested catalysts showed high activity, with chemo- and diastereo-selectivity (Table 2, entries 2–9). Among simple cinchona alkaloids C2–C5, cinchonine C4 showed the best enantioselectivity of 56% ee (entries 2–5). Introducing benzoyl and thiourea moieties at the hydroxy group of cinchonine did not improve the enantioselectivity (entries 6 and 7). The use of dimeric cinchona alkaloids C8 and C9 also led to decreased enantioselectivity (entries 8 and 9). Therefore, we selected cinchonine as the best catalyst at present. Optimization of solvents revealed that the reaction showed higher enantioselectivity (73% ee, entry 12) in acetonitrile.

**Figure 3:**
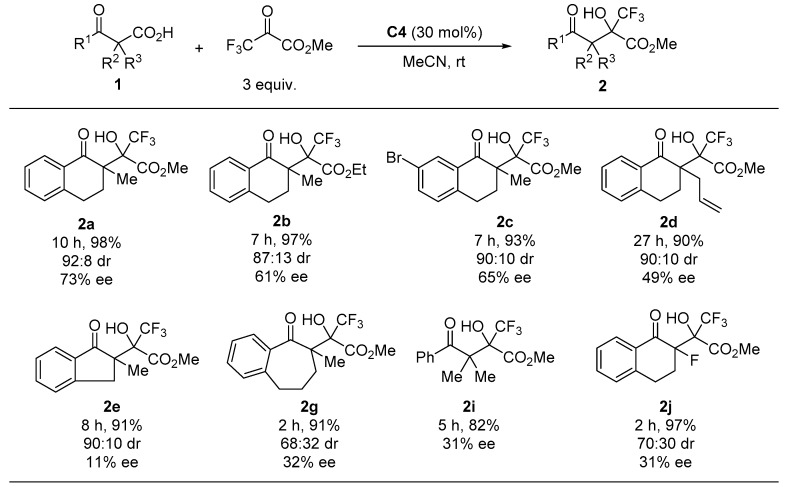
Enantioselective decarboxylative aldol reaction of 1 ^1^. ^1^ All reactions were carried out at room temperature in acetonitrile with 3 equiv. of trifluoropyruvates in the presence of 30 mol% C4. Isolated yields were described. Diastereomeric ratios were determined by ^1^H-NMR analyses. Enantiomeric excess was determined by chiral HPLC analysis.

Finally, we performed enantioselective decarboxylative aldol reactions with several β-ketocarboxylic acids (Figure 3). The use of ethyl trifluoropyruvate caused a slight decrease in both the diastereoselectivity and enantioselectivity (see **2b**). The reaction of 7-bromotetralone-derived β-ketocarboxylic acid 1c with methyl trifluoropyruvate gave the corresponding product with high diastereoselectivity and good enantioselectivity. Introducing allyl and fluoro groups at the α-position of the keto carbonyl group of the substrate significantly decreased the enantioselectivity (2d and 2i). The use of indanone- and benzosuberone-derived substrates 1e and 1g, as well as acyclic substrate 1i, resulted in poor enantioselectivity.

In conclusion, we revealed that the decarboxylative aldol reaction of tertiary β-ketocarboxylic acids with trifluoropyruvates proceeded smoothly in the presence of a tertiary amine catalyst to afford the corresponding aldol product with high diastereoselectivity. We also examined a catalytic enantioselective reaction examined using cinchonine as the catalyst to obtain the product with moderate-to-good enantioselectivity (up to 73% ee). This was the first example of the enantioselective decarboxylative aldol reaction of tertiary β-ketocarboxylic acids to form an all-carbon quaternary stereogenic center. Since the reaction led to the simultaneous formation of a trifluoromethylated chiral carbon and an adjacent quaternary chiral carbon, it would be useful for the preparation of medicinally relevant compounds.

## 3. Materials and Methods

### 3.1. General

All non-aqueous reactions were carried out in dried glassware under an argon atmosphere and stirred using magnetic stir-plates. Thin-layer chromatography analyses were performed using pre-coated silica gel plates with a fluorescent indicator (F254) (Merck Millipore, Darmstadt, Germany). Visualization was accomplished using ultraviolet (UV) light (254 nm), phosphomolybdic acid, or p-anisaldehyde. Flash column chromatography was performed using silica gel 60 (mesh size 40–100) supplied by Kanto Chemical Co., Inc. (Tokyo, Japan). ^1^H, ^13^C, and ^19^F nuclear magnetic resonance (NMR) spectra were recorded on a JNM-ECS400 (400 MHz ^1^H, 100 MHz ^13^C, 376 MHz ^19^F) or a JNM-ECX500 (500 MHz ^1^H, 126 MHz ^13^C, 470 MHz ^19^F) instrument (JEOL Ltd., Tokyo, Japan). Chemical shift values (δ) are reported in ppm (tetramethylsilane δ 0.00 ppm for ^1^H; hexafluorobenzene δ −162.2 ppm for ^19^F; residual chloroform δ 77.0 ppm for ^13^C). Direct analyses in real time (DART) mass (positive mode) analyses were performed on a JMS-T100TD time-of-flight mass spectrometer (JEOL Ltd., Tokyo, Japan). Optical rotations were measured on a P-1030 digital polarimeter (JASCO Co., Ltd., Tokyo, Japan). Analytical high-performance liquid chromatography (HPLC) was performed on a PU1586 instrument with a MD-2018 plus diode array detector (JASCO Co., Ltd., Tokyo, Japan) using a chiral column under the conditions described below. The enantiomeric purity of the compounds was determined by HPLC analysis using chiral stationary phase columns. The ^1^H, ^13^C, and ^19^F-NMR spectra of compounds **1c** and **2** and HPLC data of compounds **2** are available in the Supplementary Material.

### 3.2. Materials

Commercial grade reagents and solvents were used without further purification unless otherwise noted. Anhydrous acetonitrile was purchased from Sigma-Aldrich (St. Louis, MO, USA). Anhydrous toluene, dichloromethane, tetrahydrofurane (THF) were purchased from Kanto Chemical Co., Inc. (Tokyo, Japan) and they were used after purification via a Glass Contour solvent dispensing system (Pure Process Technology, Nashua, NH, USA). α,α-dialkyl-β-ketocarboxylic acid 1a, 1d, 1e, 1h [29], 1f, 1g [30], 1i [45], 1j, 1k [31], catalyst C6 [46], and C7 [47] were prepared according to literature procedures.

### 3.3. Synthesis of α,α-dialkyl-β-ketocarboxylic Acid 1c

Potassium hydroxide (22.0 mmol) was added to a solution of methyl 7-bromo-2-methyl-1-oxo-1,2,3,4-tetrahydronaphthalene-2-carboxylate (660 mg, 2.2 mmol) in MeOH, H_2_O, and CH_2_Cl_2_ (6 mL, 3mL, 0.6 mL), and the mixture was stirred at ambient temperature. After the reaction completed, the reaction mixture was diluted with H_2_O and washed with ethyl acetate. The resulting aqueous layer was acidified with 1.2N HCl until the solution became pH 1.0, and then extracted with CH_2_Cl_2_. The combined organic layer was dried over Na_2_SO_4_ and concentrated. The title compound was obtained as a colorless powder (430 mg, 68% yield).

^1^H-NMR (500 MHz) δ 8.18 (d, *J* = 2.3 Hz, 1H), 7.61 (d, *J* = 8.0, 2.3 Hz, 1H), 7.14 (d, *J* = 8.0 Hz, 1H), 3.07–3.00 (m, 1H), 2.96–2.90 (m, 1H), 2.57–2.52 (m, 1H), 2.19–2.14 (m, 1H), 1.54 (m, 3H); ^13^C-NMR (126 MHz) δ195.8, 142.0, 136.7, 132.6, 131.0, 130.6, 121.0, 32.8, 25.3, 20.8; HRMS (DART): [M + NH_4_]^+^ calcd. for C_12_H_15_Br_1_N_1_O_3_, 300.02353; found, 300.02335.

### 3.4. General Procedure for the Decarboxylative Aldol Reaction

α,α-Dialkyl-β-ketocarboxylic acid 1 was added to a stirred solution of DABCO (15 mol%) and trifluoropyruvates (3 equiv.) in toluene (0.1 M). Then, the mixture was stirred at ambient temperature. The reaction mixture was directly subjected to silica gel column chromatography purification to give aldol products. Some starting compounds **1** contained 2–10% of decarboxylated by-product and used as is in the aldol reaction, because they decomposed spontaneously even at ambient temperature.

*Methyl 3,3,3-trifluoro-2-hydroxy-2-(2-methyl-1-oxo-1,2,3,4-tetrahydronaphthalen-2-yl)propanoate* (**2a**)

Following general procedure, the reaction of 1a (0.53 mmol) was stirred for 7.5 h. The crude product was purified by flash column chromatography (hexane: ethyl acetate = 10: 1) to provide 2a (92% yield 152.0 mg, 94:6 d.r.) as a colorless oil. The diastereomeric ratio of 2a was determined to be 94:6 by the analysis of ^1^H-NMR.

^1^H-NMR (500 MHz) δ 7.98 (dd, *J* = 8.0, 1.2 Hz, 1H), 7.52 (dt, *J* = 7.6, 1.2 Hz, 1H), 7.32 (t, *J* = 7.6 Hz, 1H), 7.26 (d, *J* = 8.0 Hz, 1H), 5.02 (s, 1H), 3.76 (s, 3H), 3.22–3.15 (m, 1H), 3.01–2.88 (m, 2H), 2.23–2.18 (m, 1H), 1.55 (s, 3H); ^13^C-NMR (126 MHz) δ 201.6, 169.8, 142.5, 134.2, 130.2, 128.6, 128.2, 126.9, 123.4 (q, *J* = 287.9 Hz), 80.6 (q, *J* = 27.6 Hz), 53.6, 51.5, 30.5, 25.3, 16.7; ^19^F-NMR (470 MHz) δ –71.3; HRMS (DART): [M + NH_4_]^+^ calcd. for, C_15_H_19_F_3_N_1_O_4_, 334.1266; found, 334.1267.

*Ethyl 3,3,3-trifluoro-2-hydroxy-2-(2-methyl-1-oxo-1,2,3,4-tetrahydronaphthalen-2-yl)propanoate* (**2b**)

Following general procedure, the reaction of 1a (0.49 mmol) was stirred for 9 h. The crude product was purified by flash column chromatography (hexane: ethyl acetate = 10: 1) to provide 2b (85% yield 136.6 mg, 95:5 d.r.) as a colorless oil. The diastereomeric ratio of 2b was determined to be 95:5 by the analysis of ^1^H-NMR.

^1^H-NMR (500 MHz) δ 7.99 (dd, *J* = 8.0, 1.2 Hz, 1H), 7.52 (dt, *J* = 7.6, 1.2 Hz, 1H), 7.32 (t, *J* = 7.6 Hz, 1H), 7.25 (d, *J* = 8.0 Hz, 1H), 4.95 (s, 1H), 4.23 (q, *J* = 7.3 Hz, 2H), 3.22–3.15 (m, 1H), 3.00–2.89 (m, 2H), 2.22–2.18 (m, 1H), 1.55 (s, 3H), 1.17 (t, *J* = 7.3 Hz, 3H); ^13^C-NMR (126 MHz) δ 201.2, 169.2, 142.5, 134.1, 130.3, 128.6, 128.2, 126.9, 123.5 (q, *J* = 291.5 Hz), 80.6 (q, *J* = 27.6 Hz), 63.1, 51.4, 30.6, 25.4, 16.7, 13.7; ^19^F-NMR (470 MHz) δ –71.3; HRMS (DART): [M + H]^+^ calcd. for, C_16_H_18_F_3_O_4_, 331.1157; found, 331.1156.

*Methyl 2-(7-bromo-2-methyl-1-oxo-1,2,3,4-tetrahydronaphthalen-2-yl)-3,3,3-trifluoro-2-hydroxypropanoate* (**2c**)

Following general procedure, the reaction of 1c (0.18 mmol) was stirred for 4 h. The crude product was purified by flash column chromatography (hexane: ethyl acetate = 10: 1) to provide 2c (88% yield 59.8 mg, 92:8 d.r.) as a colorless oil. The diastereomeric ratio of 2c was determined to be 92:8 by the analysis of ^1^H-NMR.

^1^H-NMR (500 MHz) δ 8.01 (d, *J* = 1.9 Hz, 1H), 7.62 (dd, *J* = 8.0, 1.9 Hz, 1H), 7.15 (d, *J* = 8.0 Hz, 1H), 4.83 (s, 1H), 3.79 (s, 3H), 3.14–3.06 (m, 1H), 2.97–2.86 (m, 2H), 2.22–2.18 (m, 1H), 1.53 (s, 3H); ^13^C-NMR (126 MHz) δ 200.1, 169.7, 141.1, 136.9, 131.7, 130.9, 130.3, 123.3 (q, *J* = 289.1 Hz), 120.9, 80.4 (q, *J* = 27.6 Hz), 53.8, 51.4, 30.3, 24.9, 16.6; ^19^F-NMR (470 MHz) δ −71.4; HRMS (DART): [M + H]^+^ calcd. for, C_15_H_15_BrF_3_O_4_, 395.0106; found, 395.0105.

*Methyl 2-(2-allyl-1-oxo-1,2,3,4-tetrahydronaphthalen-2-yl)-3,3,3-trifluoro-2-hydroxypropanoate* (**2d**)

Following general procedure, the reaction of 1d (0.32 mmol) including 10% of decarboxylative product was stirred for 2 h. The crude product was purified by flash column chromatography (hexane: ethyl acetate = 10: 1) to provide 2d (88% yield 154.7 mg, 92:8 d.r.) as a pale yellow oil. The diastereomeric ratio of 2d was determined to be 92:8 by the analysis of ^1^H-NMR.

^1^H-NMR (500 MHz) δ 7.97–7.95 (m, 1H), 7.51 (m, 1H), 7.31 (t, *J* = 7.6 Hz, 1H), 7.23 (d, *J* = 7.6 Hz, 1H), 5.85–5.77 (m, 1H), 5.00 (s, 1H), 4.98–4.93 (m, 2H), 3.72 (s, 3H), 3.25–3.18 (m, 1H), 2.99–2.93 (m, 2H), 2.84–2.78 (m, 1H), 2.70–2.65 (m, 1H), 2.34–2.31 (m, 1H); ^13^C-NMR (126 MHz) δ 200.2, 169.9, 142.8, 134.5, 134.2, 132.0, 128.7, 128.1, 127.0, 123.4 (q, *J* = 287.9 Hz), 118.4, 81.5 (q, *J* = 27.6 Hz), 54.6, 53.7, 36.6, 29.0 (d, *J* = 3.6 Hz), 25.4; ^19^F-NMR (470 MHz) δ −70.4; HRMS (DART): [M + H]^+^ calcd. for, C_17_H_18_F_3_O_4_, 343.1157; found, 343.1159.

*Methyl 3,3,3-trifluoro-2-hydroxy-2-(2-methyl-1-oxo-2,3-dihydro-1H-inden-2-yl)propanoate* (**2e**)

Following general procedure, the reaction of 1e (0.38 mmol) including 9% of decarboxylative product was stirred for 3 h. The crude product was purified by flash column chromatography (hexane: ethyl acetate = 20: 3) to provide 2e (90% yield 108.8 mg, 95:5 d.r.) as a colorless oil. The diastereomeric ratio of 2e was determined to be 95:5 by the analysis of ^1^H-NMR.

^1^H-NMR (500 MHz) δ 7.77 (d, *J* = 7.6 Hz, 1H), 7.67 (m, 1H), 7.47 (d, *J* = 8.0 Hz, 1 H), 7.42 (t, *J* = 7.5 Hz, 1H), 5.32 (s, 1H), 3.84 (d, *J* = 17.6 Hz, 1H), 3.59 (s, 3H), 2.97 (d, *J* = 17.6 Hz, 1H), 1.47 (s, 3H); ^13^C-NMR (126 MHz) δ 208.3, 168.5, 152.4, 136.1, 133.5, 127.9, 126.5, 124.8, 123.0 (q, *J* = 287.9 Hz), 80.4 (q, *J* = 28.8 Hz), 53.3, 51.6, 38.3, 20.6; ^19^F-NMR (470 MHz) δ −72.0; HRMS (DART): [M + H]^+^ calcd. for, C_14_H_14_F_3_O_4_, 303.0844; found, 303.0846.

*Methyl 3,3,3-trifluoro-2-hydroxy-2-(5-methoxy-2-methyl-1-oxo-2,3-dihydro-1H-inden-2-yl)propanoate* (**2f**)

Following general procedure, the reaction of 1f (0.29 mmol) including 6% of decarboxylative product was stirred for 5 h. The crude product was purified by flash column chromatography (hexane: ethyl acetate = 10: 1) to provide 2f (82% yield 79.7 mg, 92:8 d.r.) as a colorless oil. The diastereomeric ratio of 2f was determined to be 92:8 by the analysis of ^1^H-NMR.

^1^H-NMR (500 MHz) δ 7.70 (d, *J* = 8.4 Hz, 1H), 6.94 (dd, *J* = 8.4, 2.9 Hz, 1H), 6.88 (d, *J* = 1.9 Hz, 1H), 5.63 (s, 1H), 3.90 (s, 3H), 3.81 (d, *J* = 17.8, 1H), 3.56 (s, 3H), 2.93 (d, *J* = 17.8 Hz, 1H), 1.47 (d, *J* = 1.2 Hz, 3H); ^13^C-NMR (126 MHz) δ 206.7, 168.5, 166.4, 155.9, 126.6, 126.4, 123.1 (q, *J* = 287.9 Hz), 116.3, 109.4, 80.6 (q, *J* = 28.8 Hz), 55.7, 53.2, 51.4, 38.3, 20.7; ^19^F-NMR (470 MHz) δ −72.0; HRMS (DART): [M + H]^+^ calcd. for, C_15_H_16_F_3_O_5_, 333.0950; found, 333.0951.

*Methyl 3,3,3-trifluoro-2-hydroxy-2-(6-methyl-5-oxo-6,7,8,9-tetrahydro-5H-benzo[7]annulen-6-yl)propanoate* (**2g**)

Following general procedure, the reaction of 1g (0.40 mmol) including 9% of decarboxylative product was stirred for 1 h. The crude product was purified by flash column chromatography (hexane: ethyl acetate = 10: 1) to provide 2g (77% yield 101.7 mg, 52:48 d.r.) as a colorless oil. The diastereomeric ratio of 2g was determined to be 52:48 by the analysis of ^1^H-NMR.

Major diastereomer; ^1^H-NMR (500 MHz) δ 7.40–7.32 (m, 1H), 7.28–7.21 (m, 2H), 7.13–7.11 (m, 1H), 4.49 (s, 1H), 3.90 (s, 3H), 2.87–2.85 (m, 2H), 2.61–2.57 (m, 1H), 2.06–1.92 (m, 3H), 1.42 (s, 3H); ^13^C-NMR (126 MHz) δ 209.8, 170.2, 140.2, 137.1, 130.7, 128.5, 128.0, 126.5, 123.8 (q, *J* = 289.1 Hz), 79.9 (q, *J* = 27.6 Hz), 57.8, 53.9, 34.0, 32.3, 23.2, 18.7; ^19^F-NMR (470 MHz) δ; –70.1; Minor diastereomer; ^1^H-NMR (500 MHz) δ 7.40–7.32 (m, 1H), 7.28–7.21 (m, 2H), 7.13–7.11 (m, 1H), 4.52 (s, 1H), 3.99 (s, 3H), 2.96–2.90 (m, 1H), 2.77–2.71 (m, 1H), 2.28–2.26 (m, 1H), 2.06–1.92 (m, 3H), 1.42 (s, 3H); ^13^C-NMR (126 MHz) δ 212.0, 170.3, 140.2, 137.3, 131.3, 128.4, 127.7, 126.5, 123.8 (q, *J* = 289.1 Hz), 81.4 (q, *J* = 27.6 Hz), 56.2, 54.3, 32.2, 30.4, 22.8, 19.5; ^19^F-NMR (470 MHz) δ −70.5; HRMS (DART): [M + H]^+^ calcd. for, C_16_H_18_F_3_O_4_, 331.1157; found, 331.1158.

*Methyl 2-(1-benzyl-2-oxocyclohexyl)-3,3,3-trifluoro-2-hydroxypropanoate* (**2h**)

Following general procedure, the reaction of 1h (0.88 mmol) including 2% of decarboxylative product was stirred for 2 h. The crude product was purified by flash column chromatography (hexane: ethyl acetate = 20: 3) to provide 2h (65% yield 179.2 mg, 63:37 d.r.) as a colorless oil. The diastereomeric ratio of 2h was determined to be 63:37 by the analysis of ^1^H-NMR.

Major diastereomer; ^1^H-NMR (500 MHz) δ 7.27–7.08 (m, 5H), 3.84 (s, 3H), 3.64 (d, *J* = 14.1 Hz, 1 H), 3.03 (d, *J* = 14.1 Hz, 1H), 2.59–2.41 (m, 1H), 2.38–2.27 (m,3H), 2.20–2.12 (m, 1H), 1.84–1.23 (m, 4H); ^13^C-NMR (126 MHz) δ 215.1, 170.0, 137.2, 130.8, 128.3, 126.9, 123.3 (q, *J* = 289.1 Hz), 82.0 (q, *J* = 27.6 Hz), 58.4, 53.7, 42.3, 40.8, 33.2, 23.8, 21.4; ^19^F-NMR (470 MHz) δ –69.8; Minor diastereomer; ^1^H-NMR (500 MHz) δ 7.27–7.08 (m, 5H), 4.02 (s, 3H), 3.75 (d, *J* = 13.2 Hz, 1H), 2.93 (d, *J* = 14.1 Hz, 1H), 2.59–2.41 (m, 1H), 2.38–2.27 (m,3H), 2.20–2.12 (m, 1H), 1.84–1.23 (m, 4H); ^13^C-NMR (126 MHz) δ 214.1, 169.9, 136.7, 130.7, 128.5, 126.9, 123.3 (q, *J* = 289.1 Hz), 81.0 (q, *J* = 27.6 Hz), 59.4, 54.2, 42.8, 41.9, 30.7, 22.0, 21.5; ^19^F-NMR (470 MHz) δ −69.5; HRMS (DART): [M + NH_4_]^+^ calcd. for, C_17_H_23_F_3_N_1_O_4_, 362.1579; found, 362.1578.

*Methyl 2-hydroxy-3,3-dimethyl-4-oxo-4-phenyl-2-(trifluoromethyl)butanoate* (**2i**)

Following general procedure, the reaction of 1i (0.52 mmol) was stirred for 4.5 h. The crude product was purified by flash column chromatography (hexane: ethyl acetate = 6: 1) to provide 2i (71% yield 112.5 mg) as a colorless oil.

^1^H-NMR (500 MHz) δ 7.52–7.38 (m, 5H), 4.57 (s, 1H), 3.96 (s, 3H), 1.60 (dd, *J* = 33.8, 1.0 Hz, 6H); ^13^C-NMR (126 MHz) δ 209.1, 170.2, 138.4, 128.0, 127.1, 123.7 (q, *J* = 287.9 Hz), 80.1 (q, *J* = 27.6 Hz), 55.1, 54.1, 23.5 (d, *J* = 2.4 Hz), 22.8 (d, *J* = 2.4 Hz); ^19^F-NMR (470 MHz) δ −70.6; HRMS (DART): [M + NH_4_]^+^ calcd. for, C_14_H_19_F_3_N_1_O_4_, 322.1266; found, 322.1266.

*Methyl 3,3,3-trifluoro-2-(2-fluoro-1-oxo-1,2,3,4-tetrahydronaphthalen-2-yl)-2-hydroxypropanoate* (**2j**)

Following general procedure, the reaction of 1j (0.48 mmol) was stirred for 2 h. The crude product was purified by flash column chromatography (hexane: ethyl acetate = 10: 1) to provide 2j (94% yield 144.2 mg, 77:23 d.r.) as a colorless oil. The diastereomeric ratio of 2j was determined to be 77:23 by the analysis of ^1^H-NMR.

Major diastereomer; ^1^H-NMR (500 MHz) δ 7.98 (dd, *J* = 7.6, 1.2 Hz), 7.57 (dt, *J* = 7.6, 1.2 Hz), 7.36 (t, *J* = 7.6 Hz), 7.29 (d, *J* = 7.6 Hz), 4.63 (s, 3H), 3.36–3.30 (m, 1H), 3.01 (dt, *J* = 17.2, 4.4 Hz, 1H), 2.95–2.88 (m, 1H), 2.78–2.71 (m, 1H); ^13^C-NMR (126 MHz) δ 191.2 (d, *J* = 20.4 Hz), 167.4, 143.4, 134.9, 130.3, 128.6, 128.6, 127.2, 122.3 (q, *J* = 287.9 Hz), 93.4 (d, *J* = 192.0 Hz), 79.6–78.9 (m), 54.3, 30.5 (dd, *J* = 22.8, 2.4 Hz), 24.5 (d, *J* = 7.2 Hz); ^19^F-NMR (470 MHz) δ −72.7, −172.1; Minor diastereomer; ^1^H-NMR (500 MHz), 7.99 (dd, *J* = 8.0, 0.8 Hz, 1H), 7.55 (dt, *J* = 7.6, 1.2 Hz, 1H), 7.35 (t, *J* = 7.6 Hz, 1H), 7.29 (8.0 Hz, 1H), 4.36 (s, 1H), 4.04 (s, 3H), 3.29–3.20 (m, 1H), 3.05–3.00 (m, 1H), 2.79–2.65 (m, 1H); ^13^C-NMR (126 MHz), 189.0 (d, *J* = 19.2 Hz), 169.4, 143.7, 139.4–139.1 (m), 136.8–136.6 (m), 134.8, 130.3, 128.7, 128.5, 127.2, 122.9 (q, *J* = 288.5 Hz), 93.6 (d, *J* = 196.5 Hz), 76.4–75.9 (m), 54.8, 29.4 (d, *J* = 22.0 Hz), 24.0 (d, *J* = 4.8 Hz); ^19^F-NMR (470 MHz); −72.2, −167.4; HRMS (DART): [M + NH_4_]^+^ calcd. for, C_14_H_16_F_4_N_1_O_4_, 338.1016; found, 338.1014.

*Methyl 3-benzyl-3-fluoro-2-hydroxy-4-oxo-2-(trifluoromethyl)pentanoate* (**2k**)

Following general procedure, the reaction of 1k (0.72 mmol) including 2% of decarboxylative product was stirred for 2 h. The crude product was purified by flash column chromatography (hexane: ethyl acetate = 10: 1) to provide 2k (88% yield, 203.8 mg, 75:25 d.r.) as a colorless oil. The diastereomeric ratio of 2k was determined to be 75:25 by the analysis of ^1^H-NMR.

Major diastereomer; ^1^H-NMR (500 MHz) δ 7.30–7.25 (m, 3H), 7.15–7.13 (m, 2H), 4.54 (s, 1H), 3.92 (s, 3H), 3.51–3.28 (m, 2H), 1.70 (d, *J* = 5.7 Hz, 3H); ^13^C-NMR (126 MHz) δ 208.0 (d, *J* = 30.0 Hz), 167.6, 132.5, 130.9, 128.4, 127.4, 121.1 (dq, *J* = 287.3, 2.4 Hz), 100.6 (d, *J* = 203.3 Hz), 80.1–79.1 (m), 54.4, 38.6 (d, *J* = 20.4 Hz), 28.0; ^19^F-NMR (470 MHz) δ −71.8 (d, *J* = 14.7 Hz), –166.9 (d, *J* = 44.0 Hz); Minor diastereomer; ^1^H-NMR (500 MHz) δ 7.30–7.25 (m, 3H), 7.15–7.13 (m, 2H), 4.47 (s, 1H), 4.05 (s, 3H), 3.58–3.36 (m, 2H), 1.70 (d, *J* = 5.7 Hz, 3H); ^13^C-NMR (126 MHz) δ 208.6 (d, *J* = 31.2 Hz), 166.5 (d, *J* = 6.0 Hz), 130.9, 132.5, 128.5, 127.5, 122.4 (q, *J* = 287.9 Hz), 101.5 (d, *J* = 226.3 Hz), 80.1–79.1 (m), 54.9, 39.1 (d, *J* = 22.8 Hz), 28.3; ^19^F-NMR (470 MHz) δ −71.9 (d, *J* = 14.7 Hz), −168.7 (d, *J* = 44.0 Hz); HRMS (DART): [M + NH_4_]^+^ calcd. for, C_14_H_18_F_4_N_1_O_4_, 340.1172; found, 340.1173.

### 3.5. General Procedure of the Enantioselective Decarboxylative Aldol Reaction

α,α-Dialkyl-β-ketocarboxylic acid **1** was added to a stirred solution of cinchonine (30 mol%) and trifluoropyruvates (3.0 equiv.) in toluene (0.1 M). Then, the reaction mixture was stirred at ambient temperature. The mixture was purified by flash column chromatography on silica gel to give aldol products. When the reactions were performed, some starting compounds **1** contained 5–33% of decarboxylated by-product as an impurity, as noted in the description of each compound, because some of 1 decomposed slowly while standing at ambient temperature.

*Methyl 3,3,3-trifluoro-2-hydroxy-2-(2-methyl-1-oxo-1,2,3,4-tetrahydronaphthalen-2-yl)propanoate* (**2a**)

Following general procedure, the reaction of 1a (0.30 mmol) was stirred for 10 h. The crude product was purified by flash column chromatography (hexane: ethyl acetate = 10: 1) to provide 2a (98% yield 151.9 mg, 92:8 d.r., 73% e.e. (major), 42% e.e. (minor)) as a colorless oil. The diastereomeric ratio of 2a was determined to be 92:8 by the analysis of ^1^H-NMR.

[α]_D_^28^ = −38.1 (*c* 0.5, CHCl_3_) (diastereomeric mixture); Major diastereomer; The enantiomeric purity of the title compound was determined by HPLC analysis (DAICEL CHIRALPAK IC-3, hexane: 2-propanol = 200: 1, flow rate = 1.0 mL/min, retention time; 37.8 min (minor enantiomer) and 44.9 min (major enantiomer)), Minor diastereomer; The enantiomeric purity of the title compound was determined by HPLC analysis (DAICEL CHIRALPAK IC-3, hexane: 2-propanol = 200: 1, flow rate = 1.0 mL/min, retention time; 22.6 min (major enantiomer), 29.4 min (minor enantiomer)).

*Ethyl 3,3,3-trifluoro-2-hydroxy-2-(2-methyl-1-oxo-1,2,3,4-tetrahydronaphthalen-2-yl)propanoate* (**2b**)

Following general procedure, the reaction of 1b (0.25 mmol) was stirred for 7 h. The crude product was purified by flash column chromatography (hexane: ethyl acetate = 10: 1) to provide 2b (97% yield 76.5 mg, 87:13 d.r., 61% e.e. (major), 36% e.e. (minor)) as a colorless oil. The diastereomeric ratio of 2b was determined to be 87:13 by the analysis of ^1^H-NMR.

[α]_D_^28^ = −26.5 (*c* 0.72, CHCl_3_) (diastereomeric mixture); Major diastereomer; The enantiomeric purity of the title compound was determined by HPLC analysis (DAICEL CHIRALPAK ID and ID-3, hexane: 2-propanol = 100: 1, flow rate = 1.0 mL/min, retention time; 52.7 min (minor enantiomer) and 57.1 (major enantiomer)), Minor diastereomer; The enantiomeric purity of the title compound was determined by HPLC analysis (DAICEL CHIRALPAK ID and ID-3, hexane: 2-propanol = 100: 1, flow rate = 1.0 mL/min, retention time; 32.7 min (major enantiomer), 45.6 min (minor enantiomer)).

*Methyl 2-(7-bromo-2-methyl-1-oxo-1,2,3,4-tetrahydronaphthalen-2-yl)-3,3,3-trifluoro-2-hydroxypropanoate* (**2c**)

Following general procedure, the reaction of 1c (0.35 mmol) was stirred for 7 h. The crude product was purified by flash column chromatography (hexane: ethyl acetate = 10: 1) to provide 2c (93% yield 124.0 mg, 90:10 d.r., 65% e.e. (major), 32% e.e. (minor)) as a colorless oil. The diastereomeric ratio of 2c was determined to be 90:10 by the analysis of ^1^H-NMR.

[α]_D_^28^ = −22.7 (*c* 1.6, CHCl_3_) (diastereomeric mixture); Major diastereomer; The enantiomeric purity of the title compound was determined by HPLC analyses (DAICEL CHIRALPAK ID-3, hexane: 2-propanol = 50: 1, flow rate = 1.0 mL/min, retention time; 19.9 min (minor enantiomer) and 23.5 min (major enantiomer), Minor diastereomer; The enantiomeric purity of the title compound was determined by HPLC analysis (DAICEL CHIRALPAK ID-3, hexane: 2-propanol = 50: 1, flow rate = 1.0 mL/min, retention time;13.5 min (major enantiomer), 17.7 min (major enantiomer)).

*Methyl 2-(2-allyl-1-oxo-1,2,3,4-tetrahydronaphthalen-2-yl)-3,3,3-trifluoro-2-hydroxypropanoate* (**2d**)

Following general procedure, the reaction of 1d (0.32 mmol) including 5% of decarboxylative product was stirred for 27 h. The crude product was purified by flash column chromatography (hexane: ethyl acetate = 10: 1) to provide 2d (90% yield 91.5 mg, 90:10 d.r., 49% e.e. (major), 9% e.e. (minor)) as a pale yellow oil. The diastereomeric ratio of 2d was determined to be 90:10 by the analysis of ^1^H-NMR.

[α]_D_^28^ = −32.3 (*c* 0.77, CHCl_3_) (diastereomeric mixture); Major diastereomer; The enantiomeric purity of the title compound was determined by HPLC analysis (DAICEL CHIRALPAK IE-3, hexane: 2-propanol = 200: 1, flow rate = 1.0 mL/min, retention time; 35.5 min (major enantiomer) and 40.2 min (minor enantiomer)), Major diastereomer; The enantiomeric purity of the title compound was determined by HPLC analysis (DAICEL CHIRALPAK IE-3, hexane: 2-propanol = 200: 1, flow rate = 1.0 mL/min, retention time; 22.0 min (major enantiomer), 30.2 min (minor enantiomer)).

*Methyl 3,3,3-trifluoro-2-hydroxy-2-(2-methyl-1-oxo-2,3-dihydro-1H-inden-2-yl)propanoate* (**2e**)

Following general procedure, the reaction of 1e (0.38 mmol) including 5% of decarboxylative product was stirred for 3 h. The crude product was purified by flash column chromatography (hexane: ethyl acetate = 20: 3) to provide 2e (91% yield 76.4 mg, 90:10 d.r., 11% e.e. (major), 26% e.e. (minor)) as a colorless oil. The diastereomeric ratio of 2e was determined to be 90:10 by the analysis of ^1^H-NMR.

Major diastereomer; The enantiomeric purity of the title compound was determined by HPLC analysis (DAICEL CHIRALPAK IE-3, hexane: 2-propanol = 200: 1, flow rate = 1.0 mL/min, retention time; 52.7 min (minor enantiomer) and 68.5 min (major enantiomer)), Minor diastereomer; The enantiomeric purity of the title compound was determined by HPLC analysis (DAICEL CHIRALPAK IE-3, hexane: 2-propanol = 200: 1, flow rate = 1.0 mL/min, retention time; 34.0 min (minor diastereomer, minor enantiomer), 43.2 min (minor diastereomer, major enantiomer)).

*Methyl 3,3,3-trifluoro-2-hydroxy-2-(6-methyl-5-oxo-6,7,8,9-tetrahydro-5H-benzo[7]annulen-6-yl)propanoate* (**2g**)

Following general procedure, the reaction of 1g (0.20 mmol) including 33% of decarboxylative product was stirred for 2 h. The crude product was purified by flash column chromatography (hexane: ethyl acetate = 10: 1) to provide 2g (91% yield 66.1 mg, 68:32 d.r., 32% e.e. (major), 16% e.e. (minor)) as a colorless oil. The diastereomeric ratio of 2g was determined to be 52:48 by the analysis of ^1^H-NMR.

Major diastereomer; The enantiomeric purity of the title compound was determined by HPLC analysis (DAICEL CHIRALPAK IB-3, hexane: 2-propanol = 200: 1, flow rate = 1.0 mL/min, retention time; 20.8 min (major enantiomer) and 33.0 min (minor enantiomer)), Minor diastereomer; The enantiomeric purity of the title compound was determined by HPLC analysis (DAICEL CHIRALPAK IB-3, hexane: 2-propanol = 200: 1, flow rate = 1.0 mL/min, retention time; 12.9 min (major enantiomer), 15.7 min (minor enantiomer)).

*Methyl 2-hydroxy-3,3-dimethyl-4-oxo-4-phenyl-2-(trifluoromethyl)butanoate* (**2i**)

Following general procedure, the reaction of 1i (0.39 mmol) was stirred for 4.5 h. The crude product was purified by flash column chromatography (hexane: ethyl acetate = 6: 1) to provide 2i (82% yield 97.3 mg, 31% e.e.) as a colorless oil.

[α]_D_^28^ = +7.6 (*c* 1.2, CHCl_3_); The enantiomeric purity of the title compound was determined by HPLC analysis (DAICEL CHIRALPAK IA-3, hexane: 2-propanol = 200: 1, flow rate = 1.0 mL/min, retention time; 24.8 min (major enantiomer), 29.5 min (minor enantiomer)).

*Methyl 3,3,3-trifluoro-2-(2-fluoro-1-oxo-1,2,3,4-tetrahydronaphthalen-2-yl)-2-hydroxypropanoate* (**2j**)

Following general procedure, the reaction of 1j (0.48 mmol) was stirred for 2 h. The crude product was purified by flash column chromatography (hexane: ethyl acetate = 10: 1) to provide 2j (97% yield 148.0 mg, 70:30 d.r., 30% e.e. (major), 31% e.e. (minor)) as a colorless oil. The diastereomeric ratio of 2j was determined to be 70:30 by the analysis of ^1^H-NMR.

Major diastereomer; [α]_D_^28^ = +21.1 (*c* 0.9, CHCl_3_); The enantiomeric purity of the title compound was determined by HPLC analysis (DAICEL CHIRALPAK IC-3, hexane: 2-propanol = 10: 1, flow rate = 1.0 mL/min, retention time; 11.1 min (major enantiomer), 17.2 min (minor enantiomer)); Minor diastereomer; [α]_D_^28^ = +6.3 (*c* 1.0, CHCl_3_); The enantiomeric purity of the title compound was determined by HPLC analysis (DAICEL CHIRALPAK IC-3, hexane: 2-propanol = 10: 1, flow rate = 1.0 mL/min, retention time; 5.7 min (major enantiomer), 6.2 min (minor enantiomer)).

## Figures and Tables

**Table 1:**
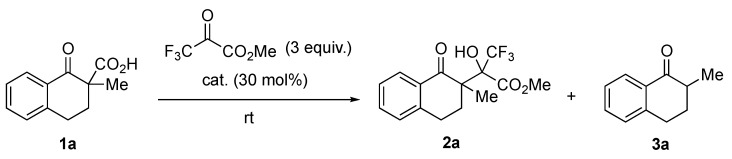
Optimization of the amine catalyst ^1^.

Entry	Catalyst	Solvent	Time (h)	Yield (%) ^2^		dr ^3^
				2a	3a	
1	none	toluene	4	<1	20	66:33
2	PhCH_2_CH_2_NH_2_	toluene	4	2	10	55:45
3	(PhCH_2_)_2_NH	toluene	4	58	10	92:8
4	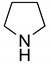	toluene	4	19	12	88:12
5	pyridine	toluene	4	6	4	68:32
6	DMAP	toluene	4	39	9	82:18
7	Et_3_N	toluene	4	96	1	86:14
8	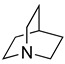 (quinuclidine)	toluene	2	98	1	93:7
9	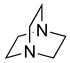 (DABCO)	toluene	2	94	3	92:8
10 ^4^	DABCO	toluene	8	92	3	94:6
11 ^4^	DABCO	MeCN	2	62	10	71:29
12 ^4^	DABCO	Et_2_O	2	47	48	74:26
13 ^4^	DABCO	CH_2_Cl_2_	2	57	9	84:16
14 ^4^	DABCO	MeOH	2	0	55	–

^1^ All reactions were carried out at room temperature with 3 equiv. of methyl trifluoropyruvate in the presence of 30 mol% catalyst, unless otherwise noted. ^2^ Isolated yield. ^3^ Determined by ^1^H-NMR analysis. ^4^ 15 mol% DABCO was used.

**Table 2:**
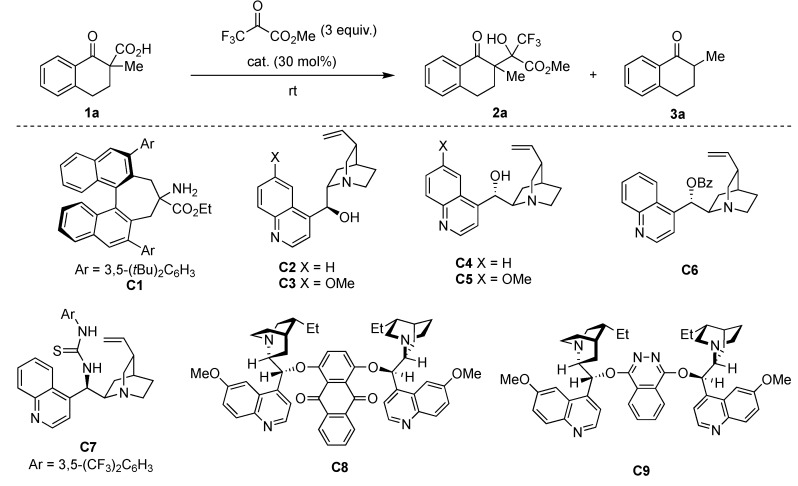
Screening of chiral amine catalyst ^1^.

Entry	Catalyst	Solvent	Time (h)	Yield (%) ^2^		dr ^3^	%ee ^4^ (Major/Minor)
				2a	3a		
1	**C1**	toluene	31	25	50	73:27	0/0
2	**C2**	toluene	10	99	0	92:8	–41/–28
3	**C3**	toluene	7	91	6	93:7	–33/–22
4	**C4**	toluene	9	89	0	94:6	50/33
5	**C5**	toluene	3	80	10	94:6	44/31
6	**C6**	toluene	5	88	3	90:10	–45/–63
7	**C7**	toluene	33	40	53	74:26	–2/0
8	**C8**	toluene	1	83	10	92:8	5/44
9	**C9**	toluene	0.5	96	4	94:6	33/58
10	**C4**	THF	4	99	0	93:7	57/35
11	**C4**	CH_2_Cl_2_	7	89	8	92:8	65/32
12	**C4**	MeCN	10	98	0	92:8	73/42

^1^ All reactions were carried out at room temperature with 3 equiv. of methyl trifluoropyruvate in the presence of 30 mol% chiral amine catalyst. ^2^ Isolated yield. ^3^ Determined by ^1^H-NMR analysis. ^4^ Determined by chiral high-performance liquid chromatography (HPLC) analysis.

## Supplementary Materials

The following are available online, ^1^H, ^13^C, and ^19^F-NMR spectra of compounds **2** and HPLC data of compound **2**.
