# Supplementary material for: Amine-Catalyzed Decarboxylative Aldol Reaction of β-Ketocarboxylic Acids with Trifluoropyruvates
Source: Molecules. 2019 Jul 30;24(15):2773. doi: 10.3390/molecules24152773 (PMC6695914; doi:10.3390/molecules24152773)
Supplement: Supplementary file 1 [file molecules-24-02773-s001.pdf]

Supplementary Materials

Amine-catalyzed Decarboxylative Aldol Reaction of β-Ketocarboxylic Acids with Trifluoropyruvates

Ryouta Kawanishi[1], Shinya Hattori[1] Seiji Iwasa[1], Kazutaka Shibatomi[1*]

[1] Department of Applied Chemistry and Life Science, Toyohashi University of Technology, 1-1 Hibarigaoka, Tempaku-cho, Toyohashi 441-8580, Japan

* Correspondence: shiba@chem.tut.ac.jp

Table of Contents

^{1}H, ^{13}C, and ^{19}F NMR spectra of compound **2**....................................2

HPLC traces of compound **2**...27

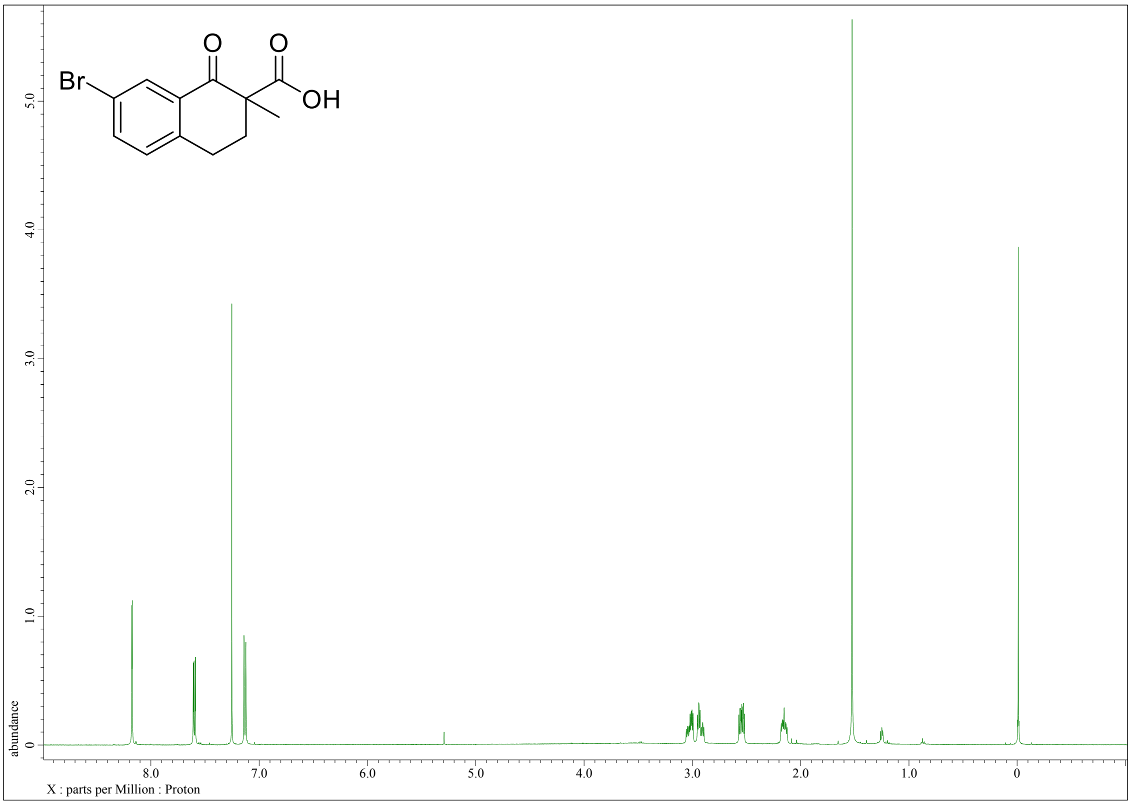

^{1}H NMR spectrum

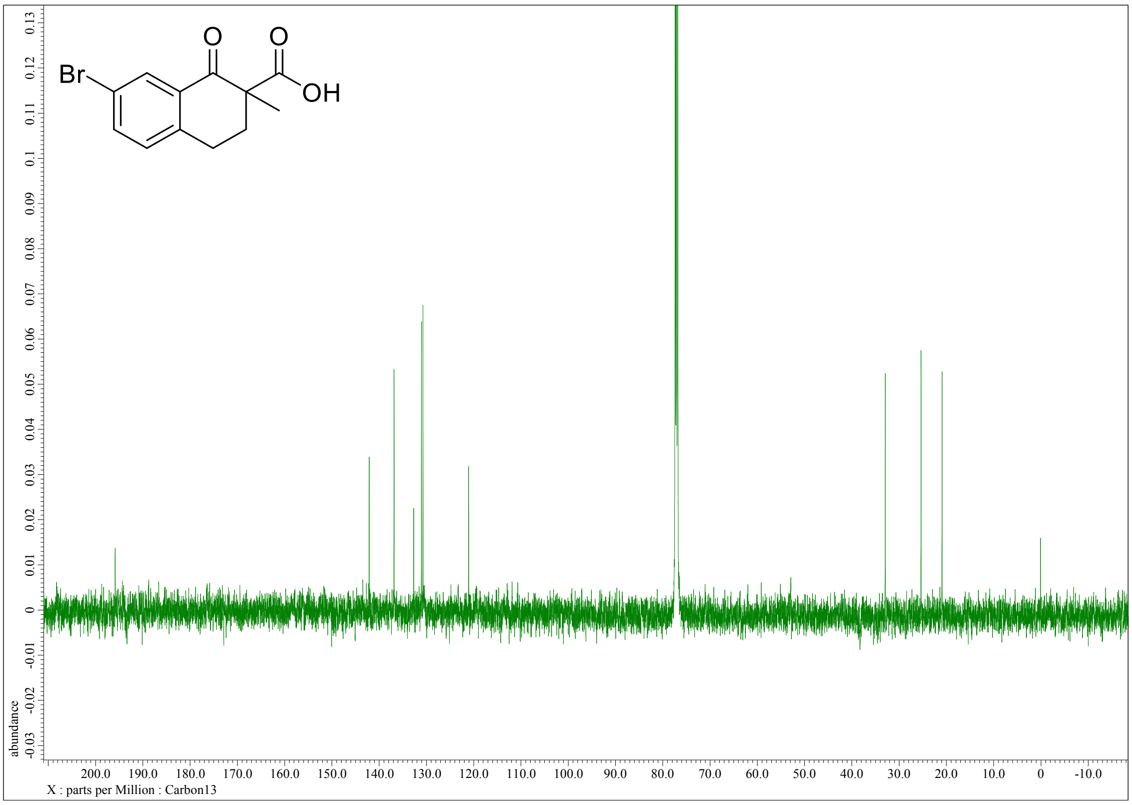

^{13}C NMR spectrum

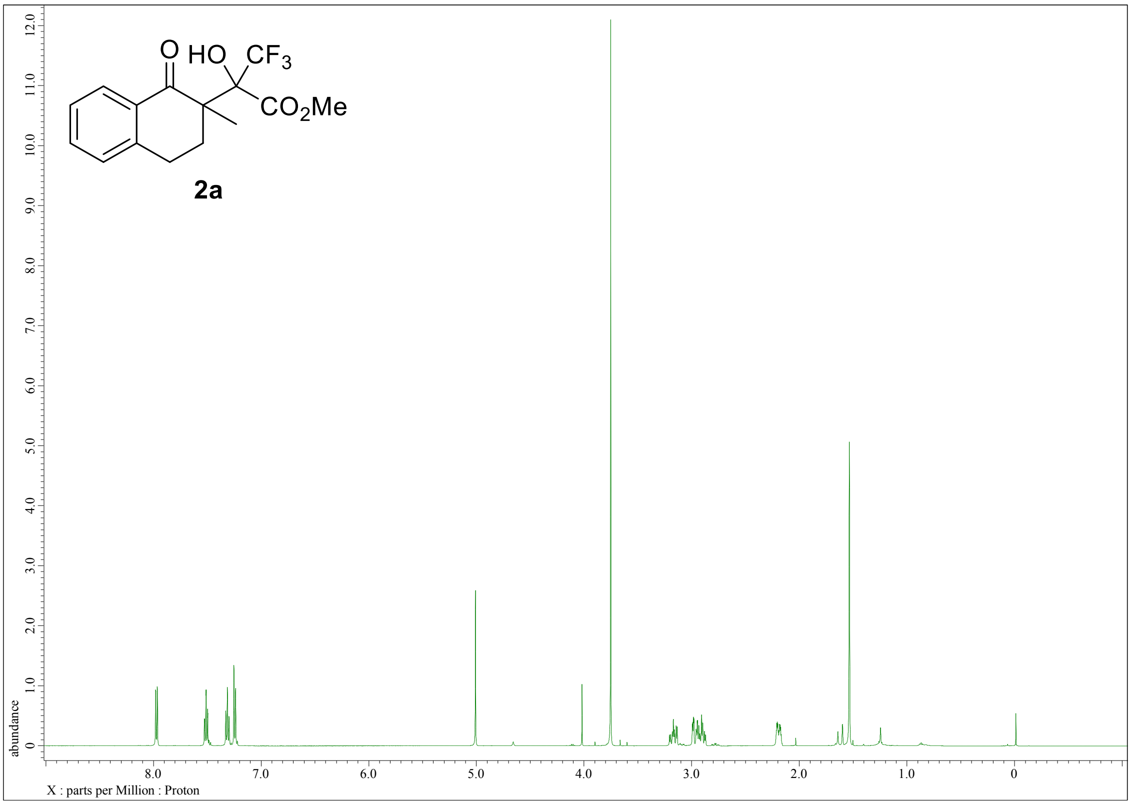

^{1}H NMR spectrum

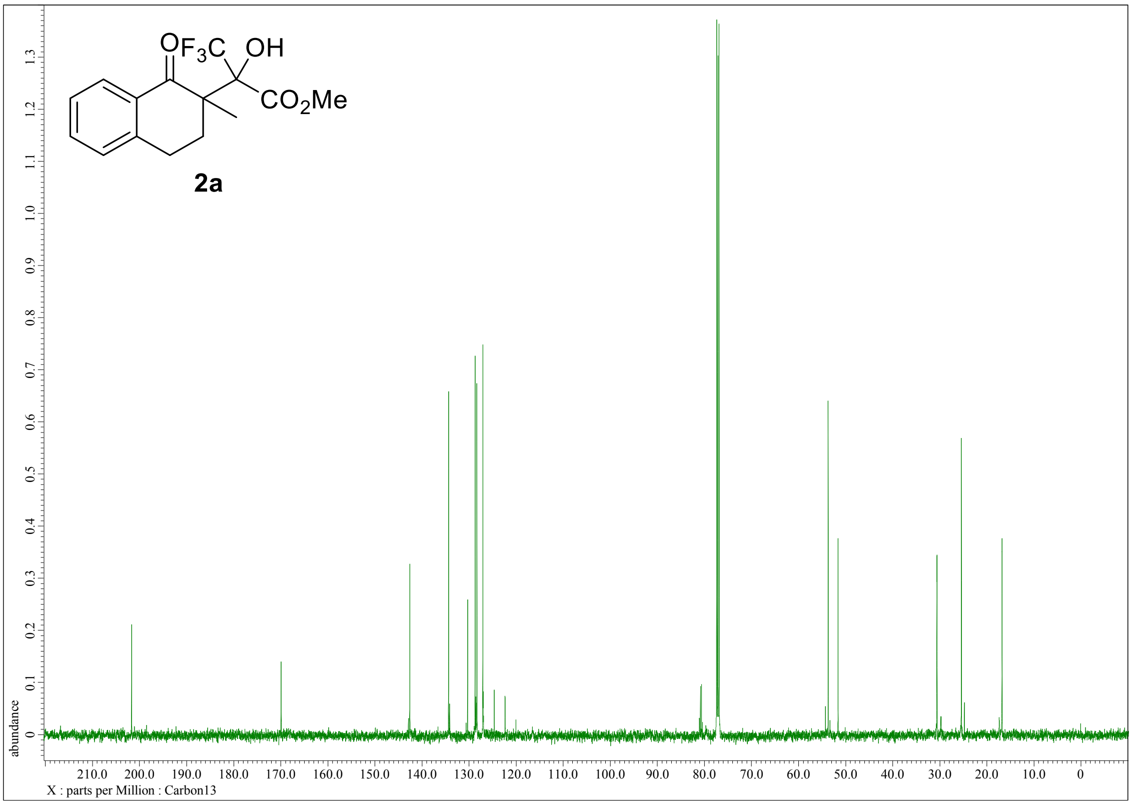

^{13}C NMR spectrum

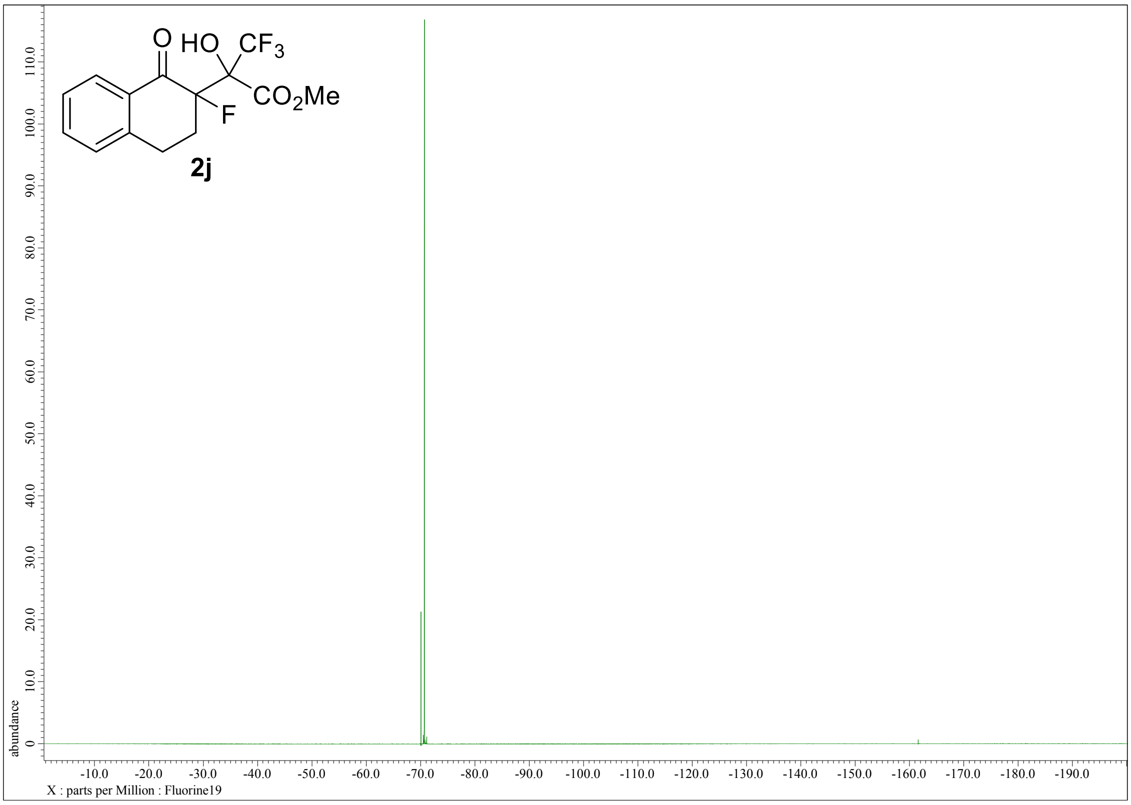

^{19}F NMR spectrum

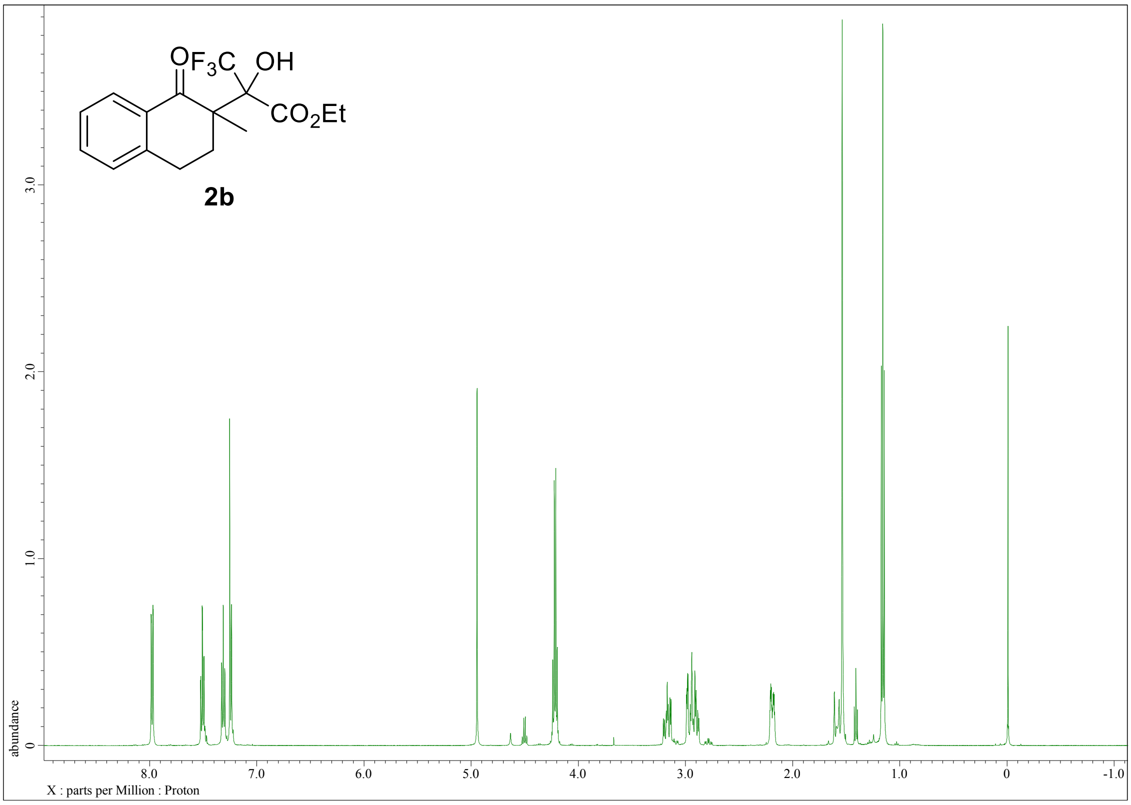

^{1}H NMR spectrum

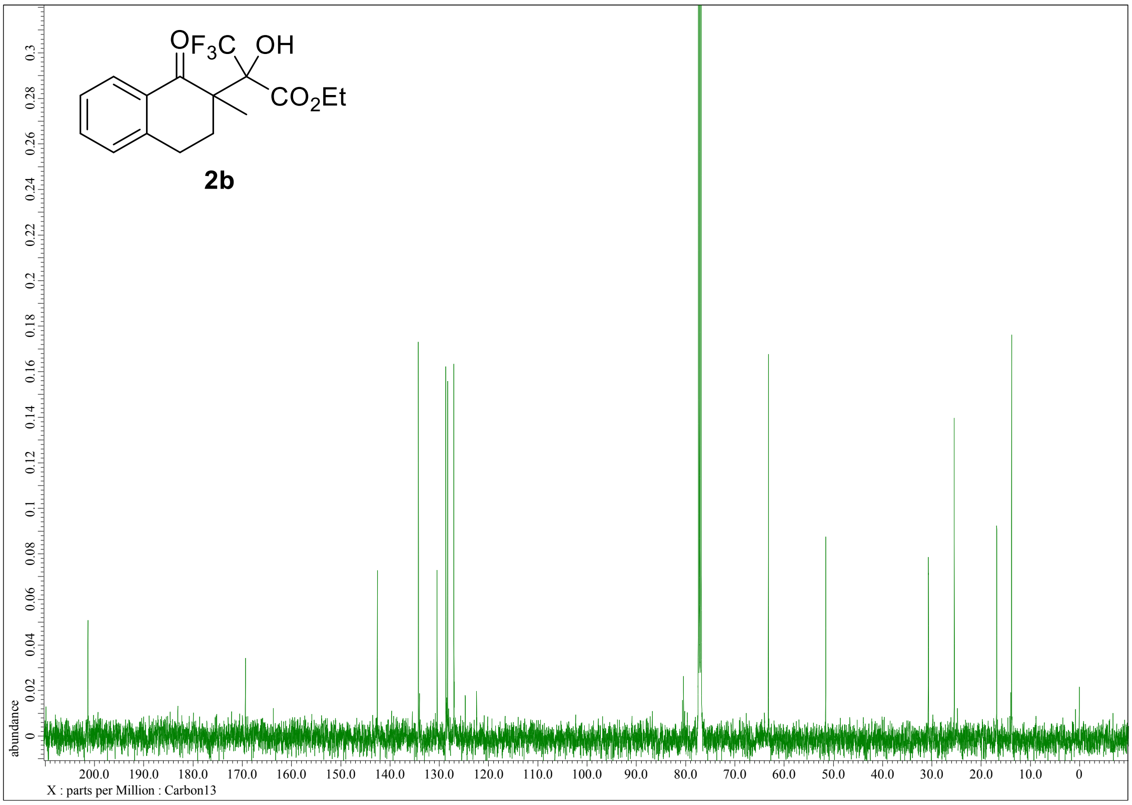

^{13}C NMR spectrum

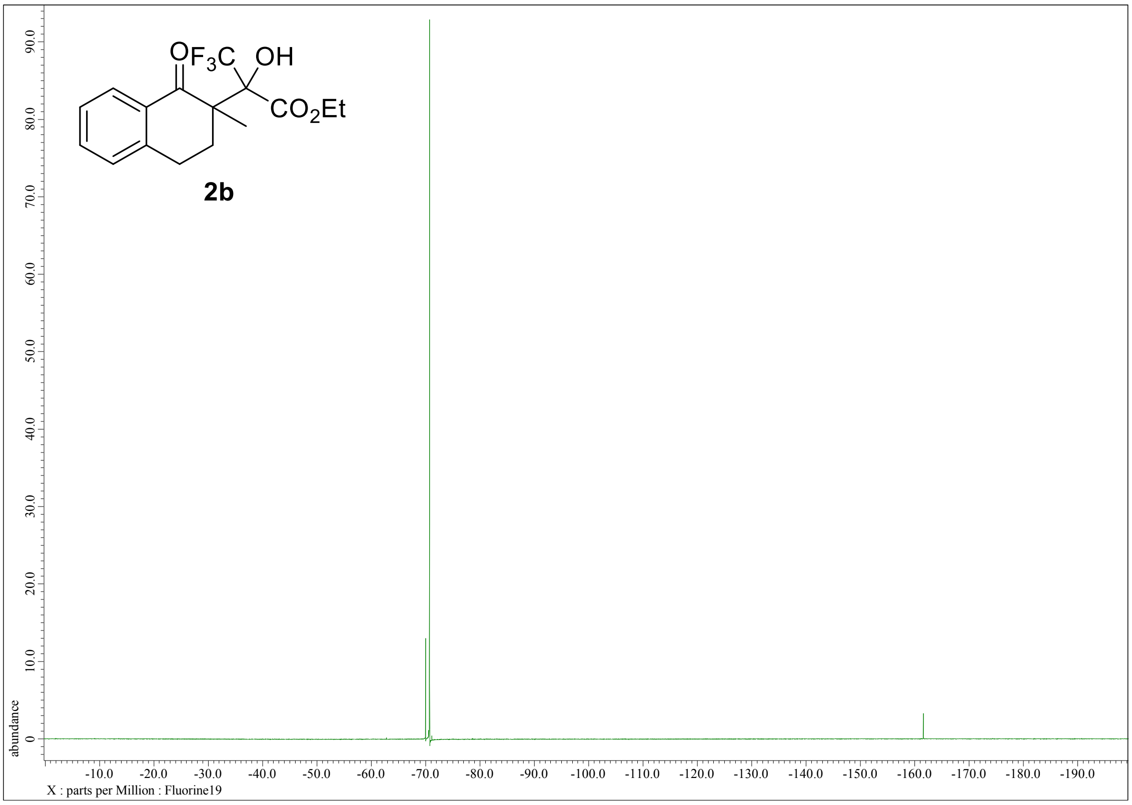

^{19}F NMR spectrum

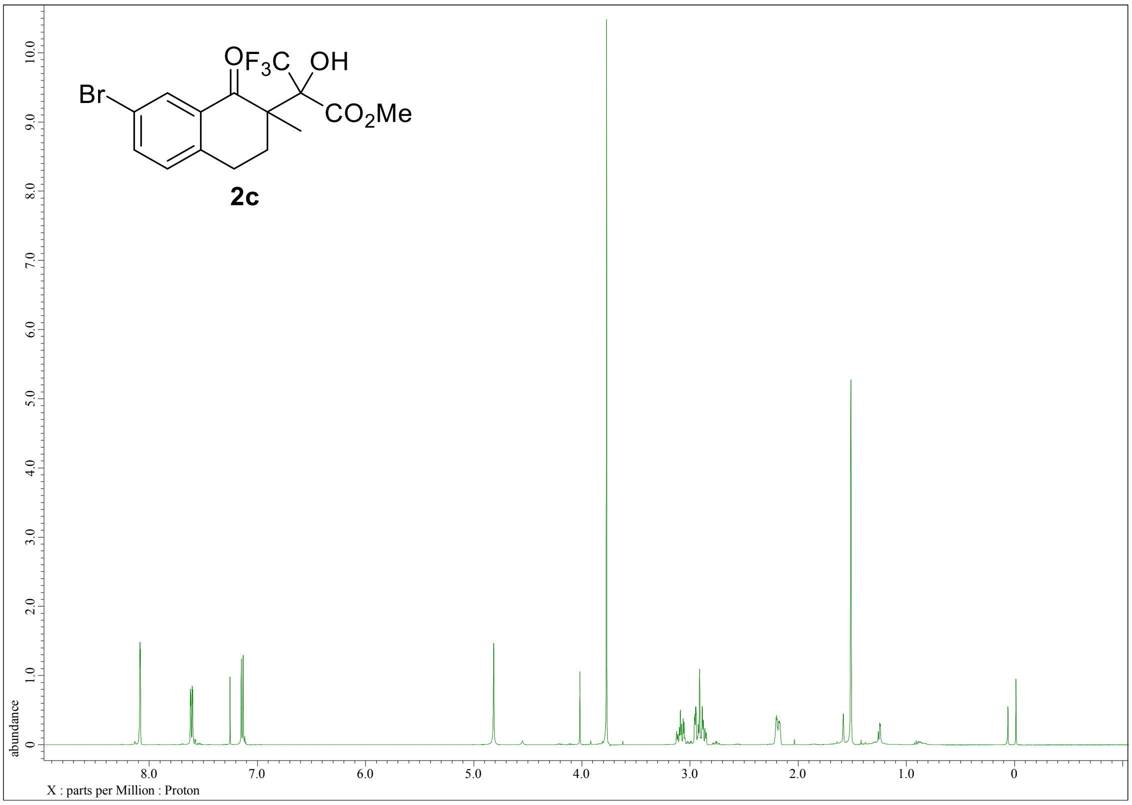

^{1}H NMR spectrum

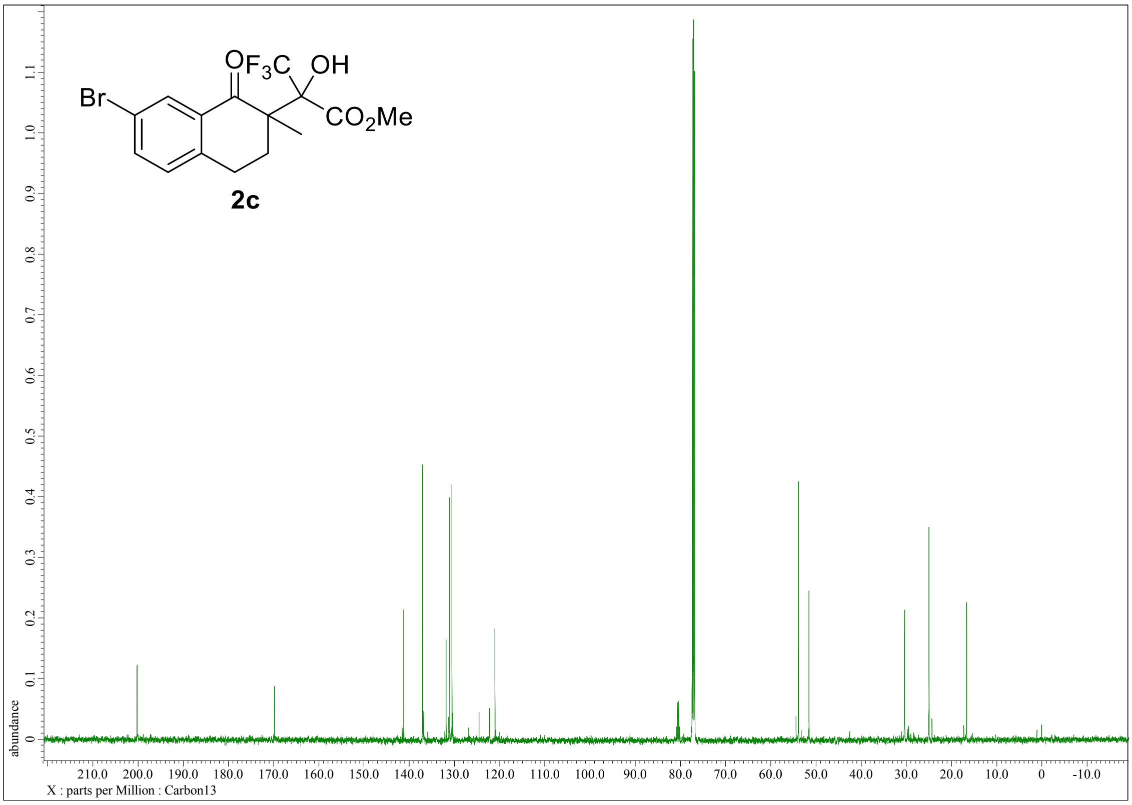

^{13}C NMR spectrum

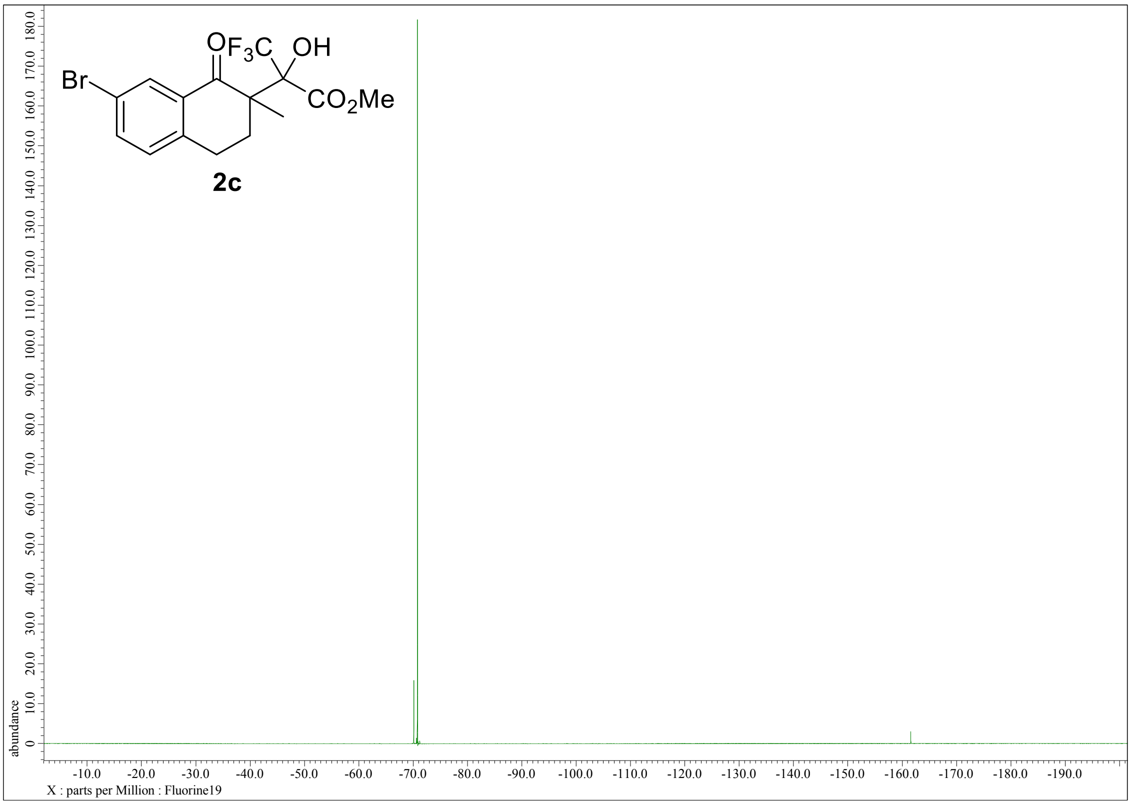

^{19}F NMR spectrum

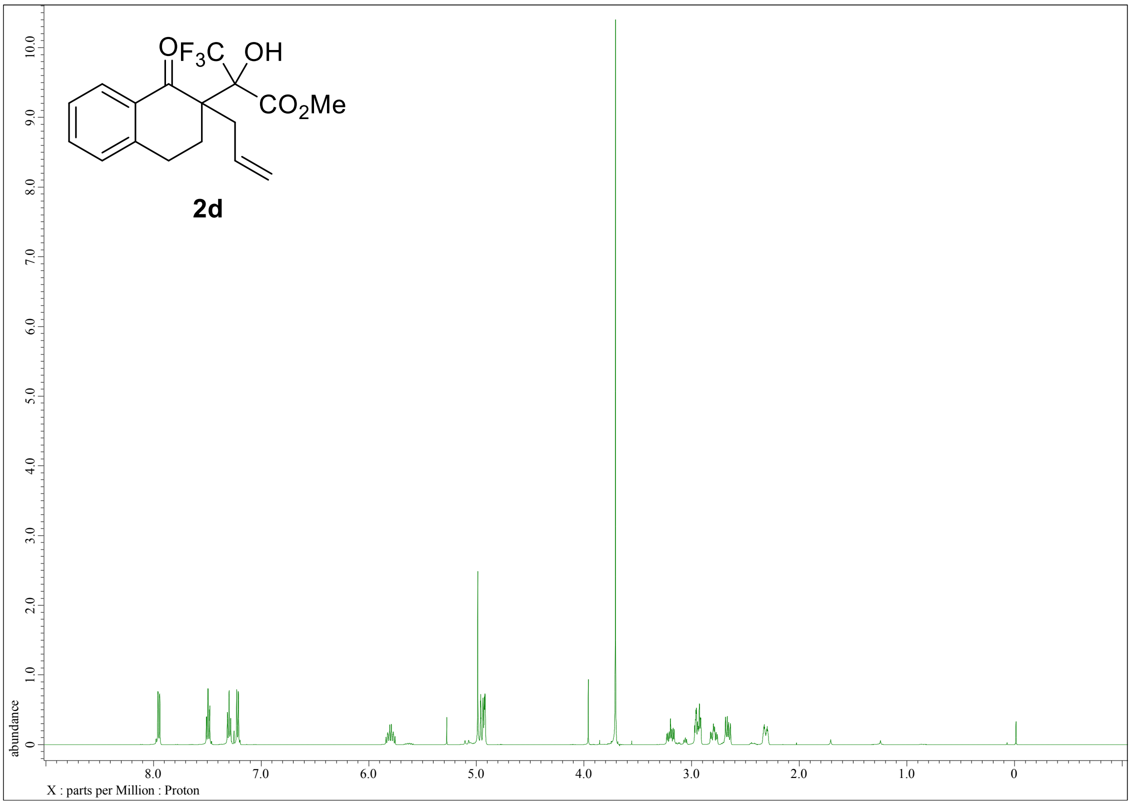

^{1}H NMR spectrum

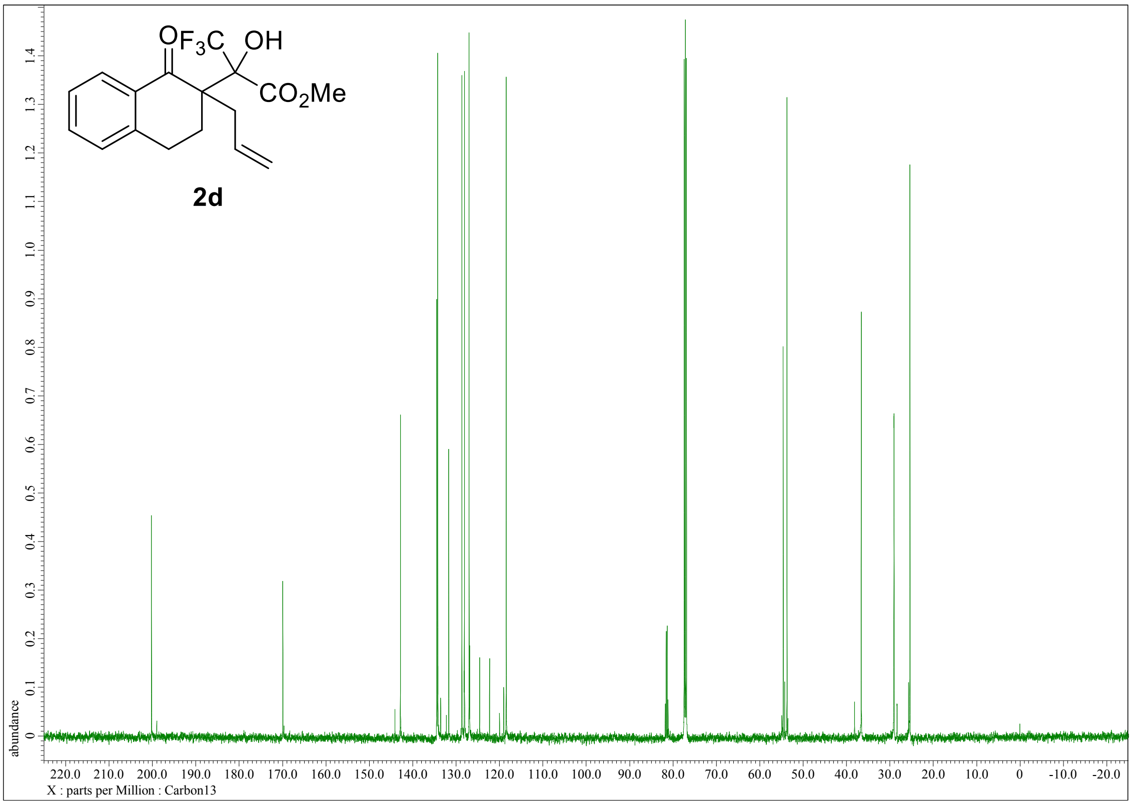

^{13}C NMR spectrum

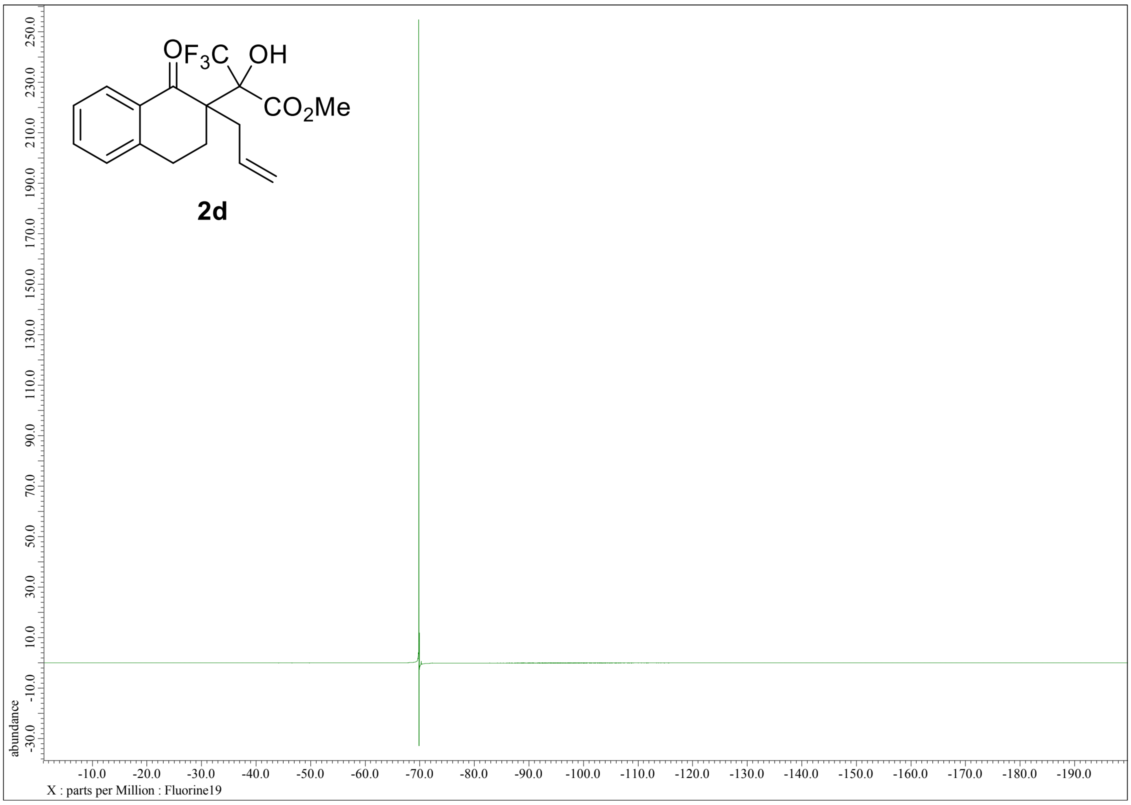

^{19}F NMR spectrum

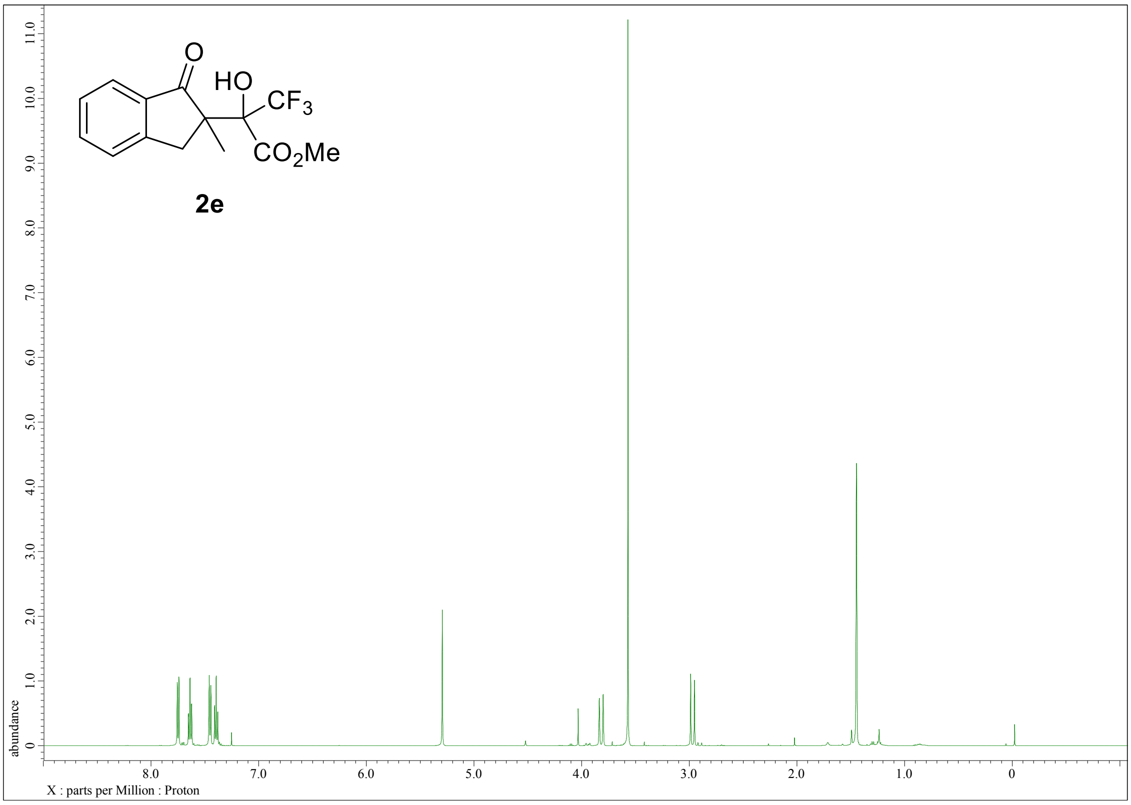

^{1}H NMR spectrum

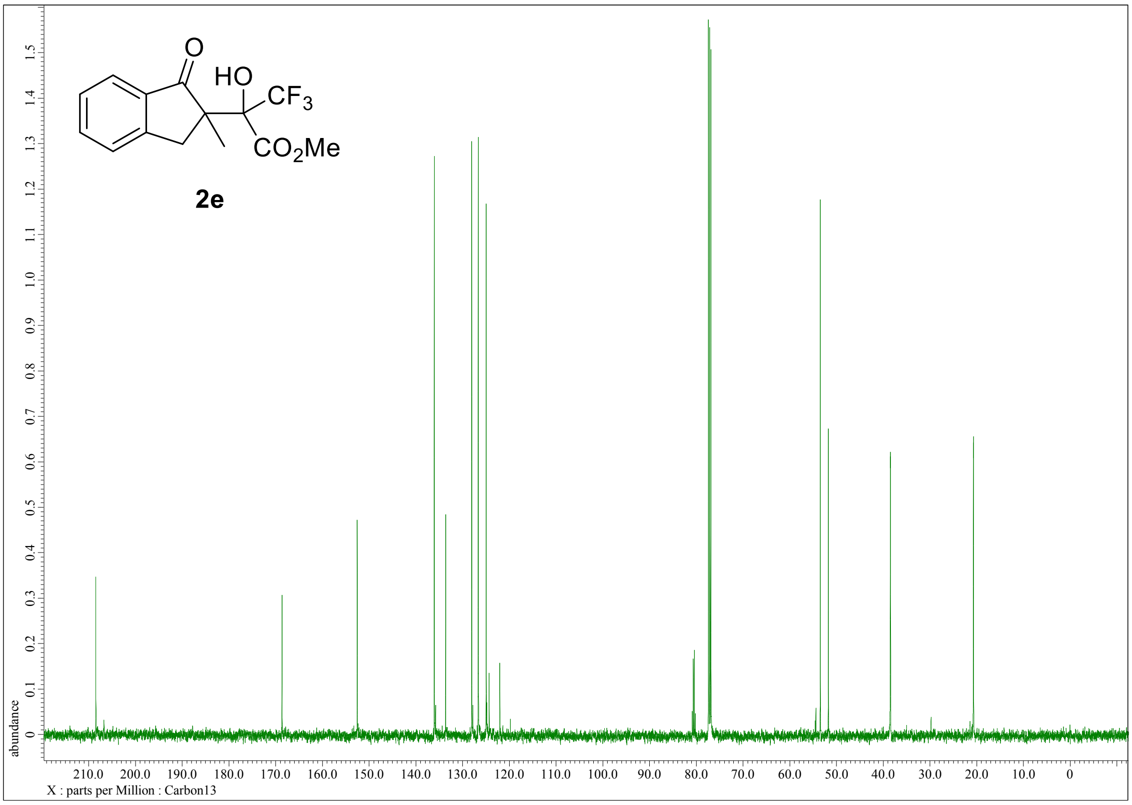

^{13}C NMR spectrum

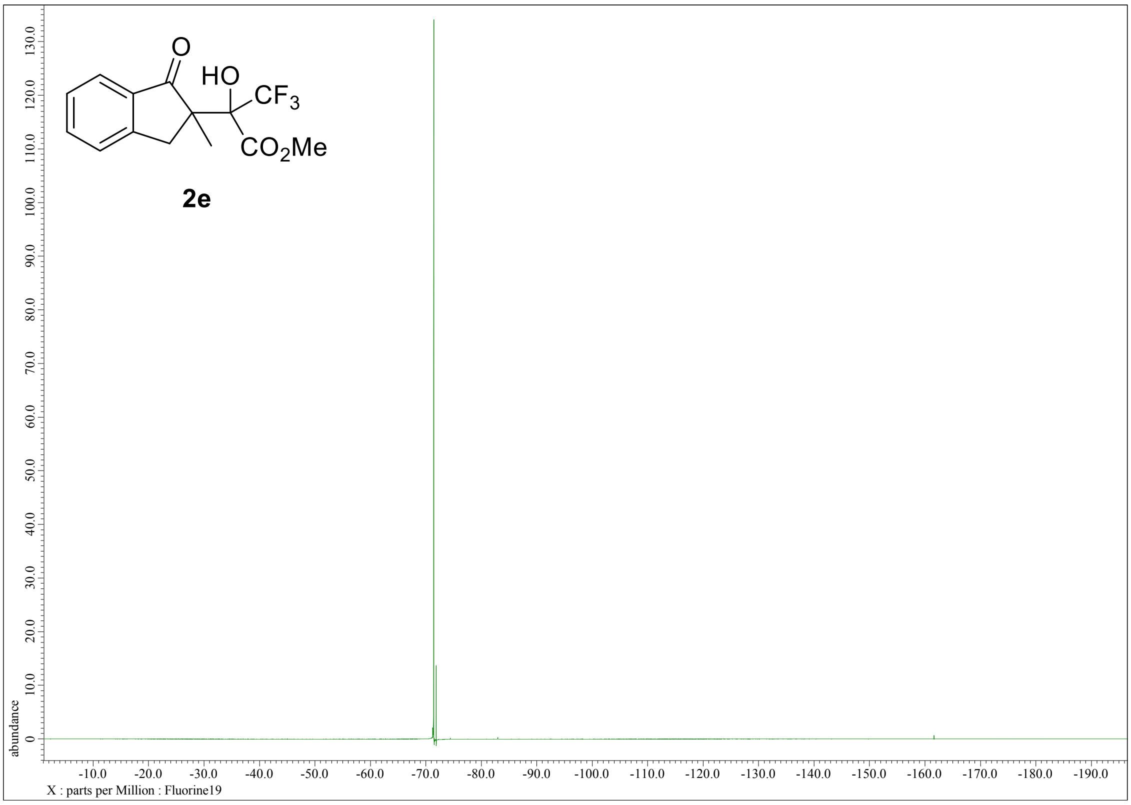

^{19}F NMR spectrum

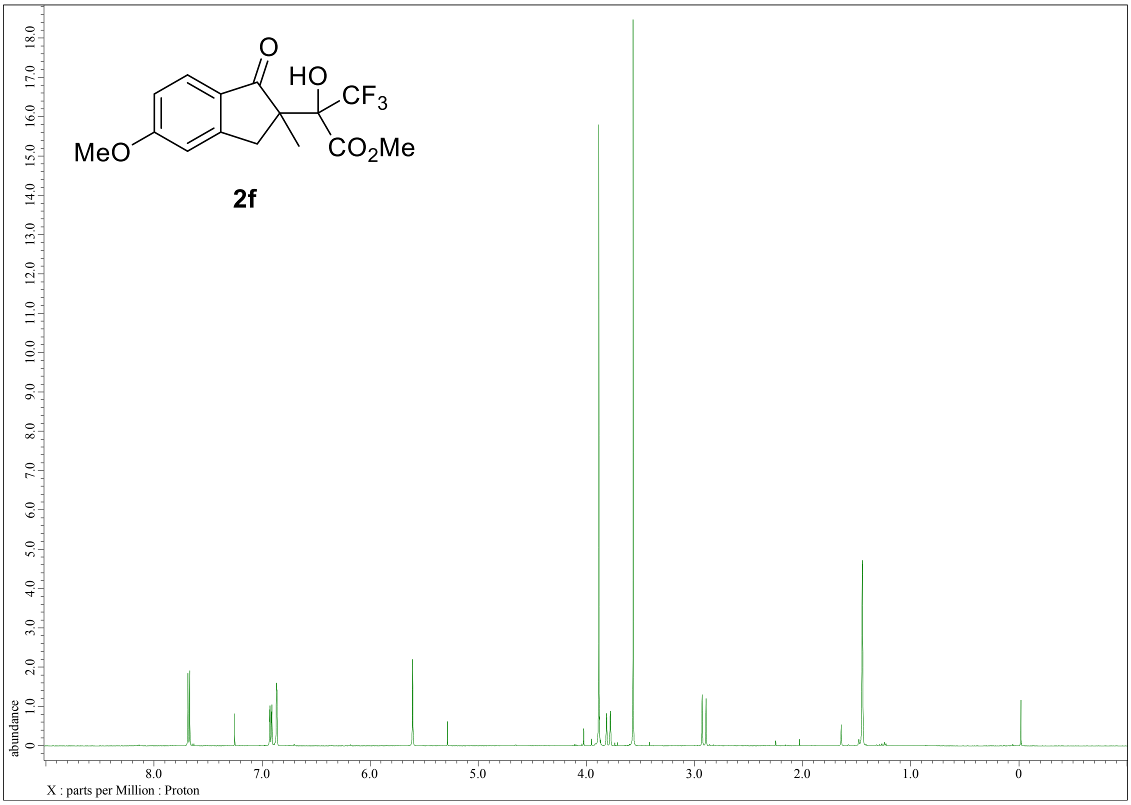

^{1}H NMR spectrum

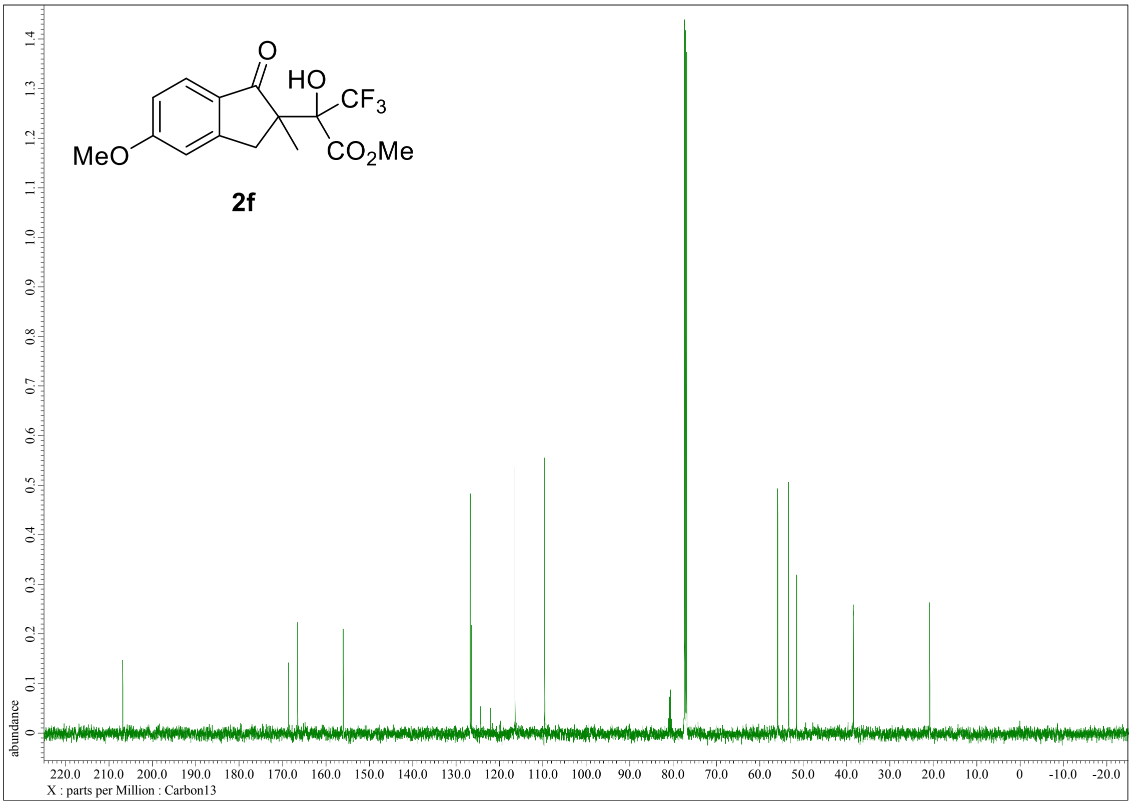

^{13}C NMR spectrum

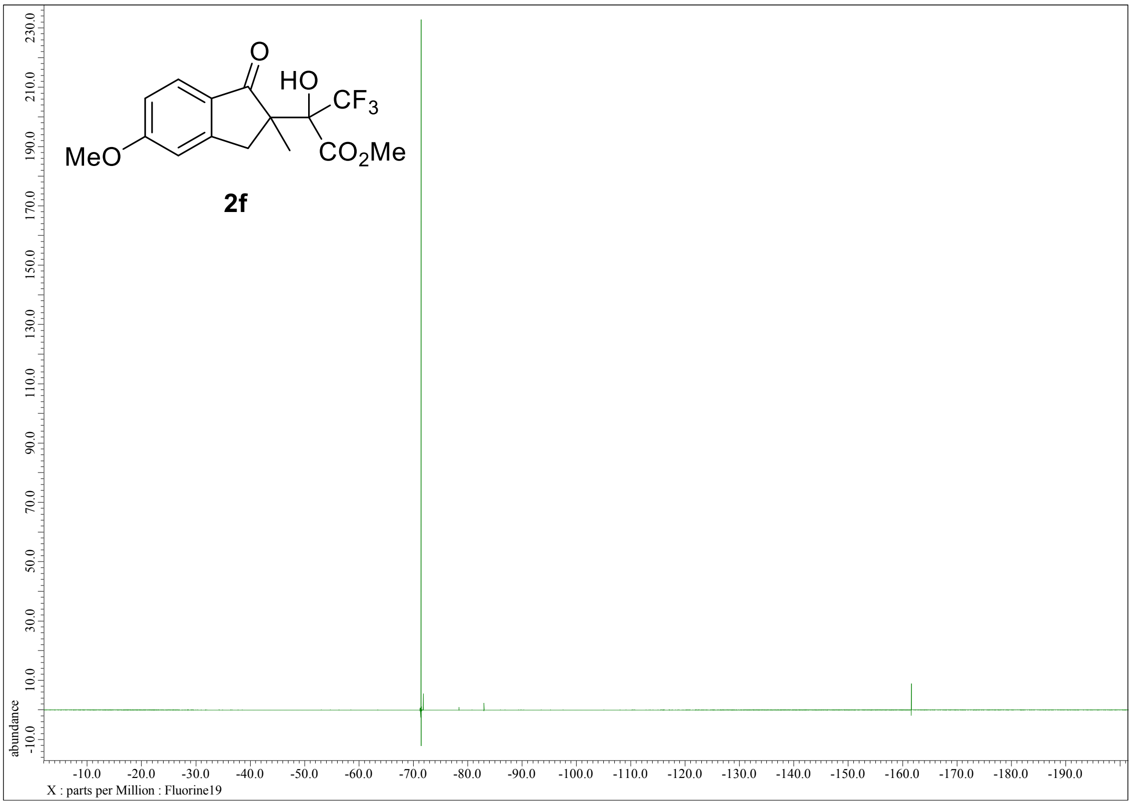

^{19}F NMR spectrum

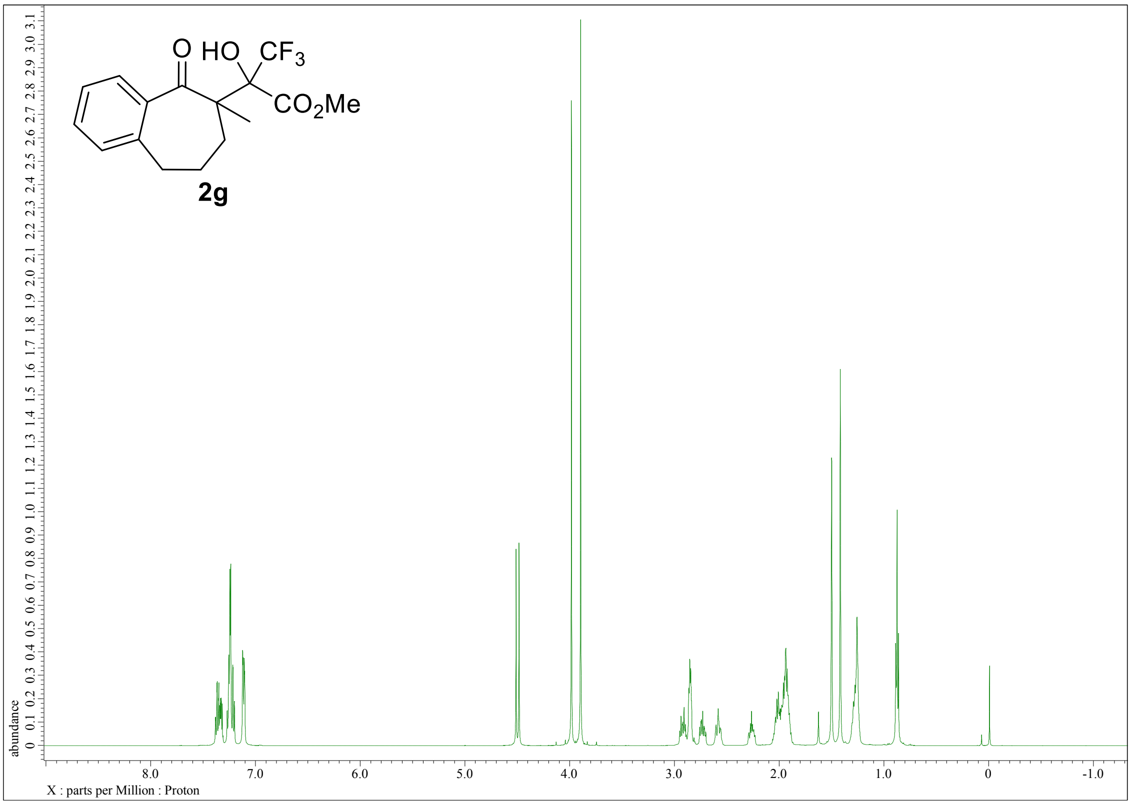

^{1}H NMR spectrum

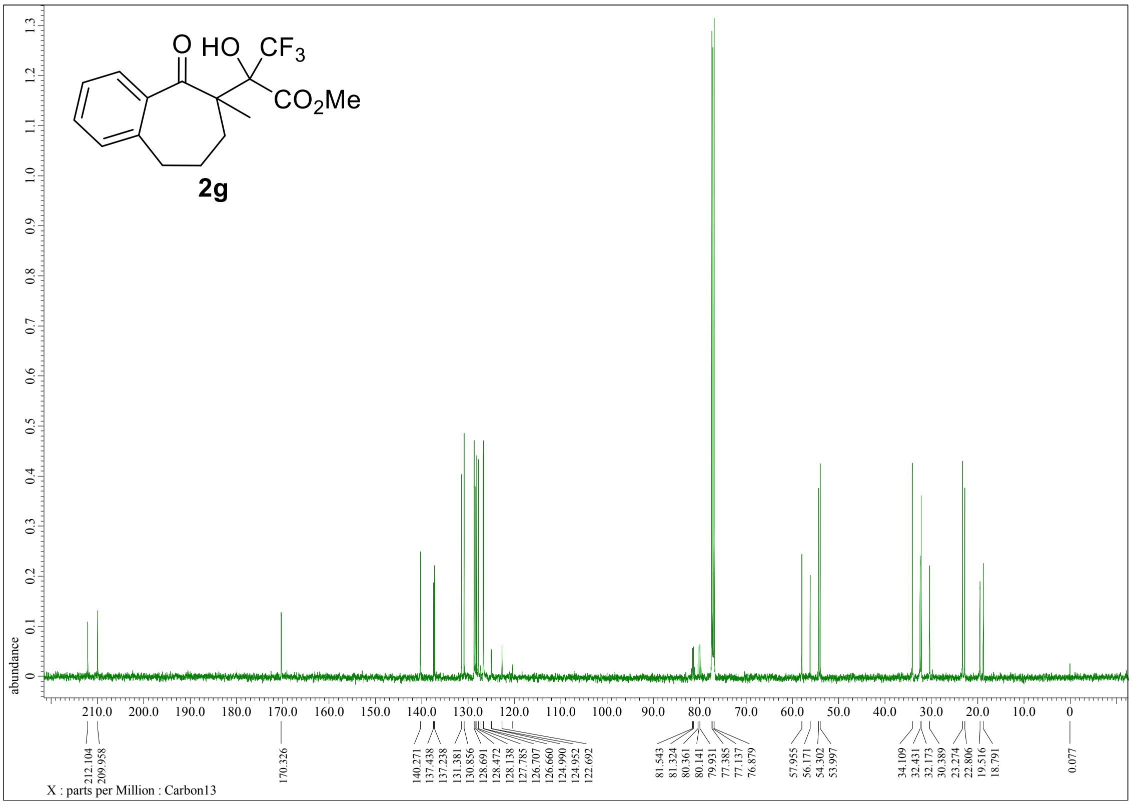

^{13}C NMR spectrum

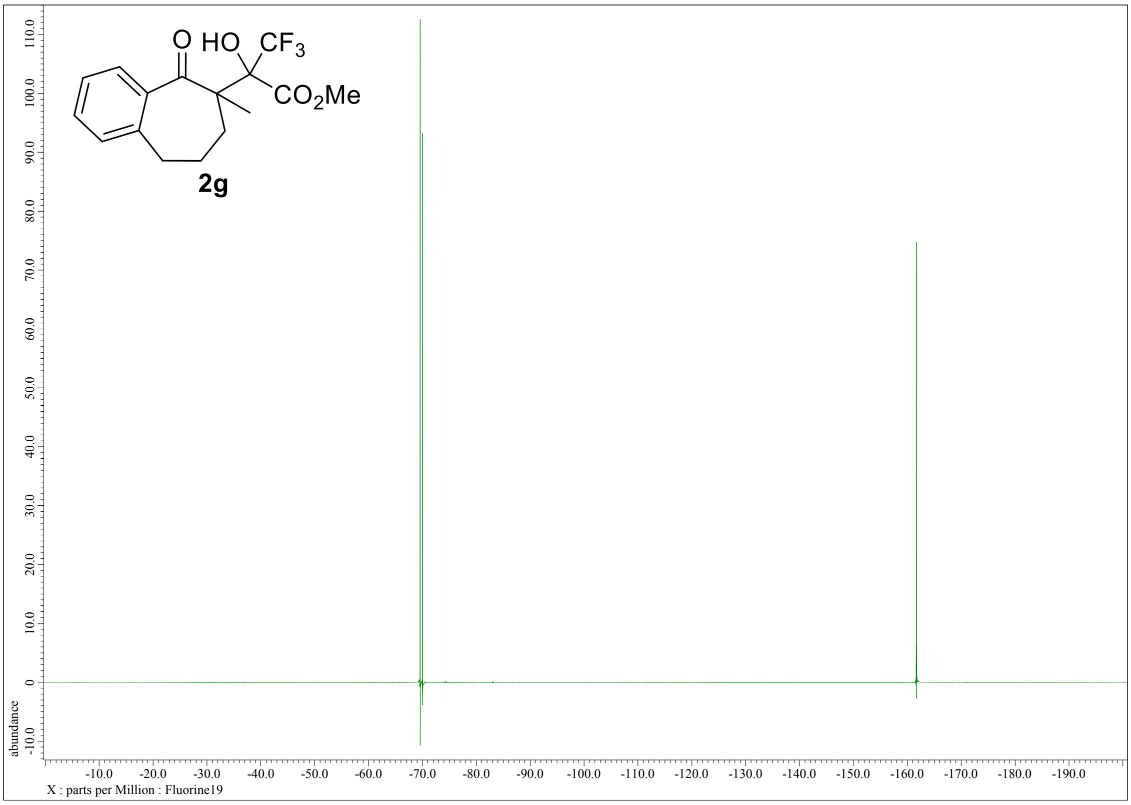

^{19}F NMR spectrum

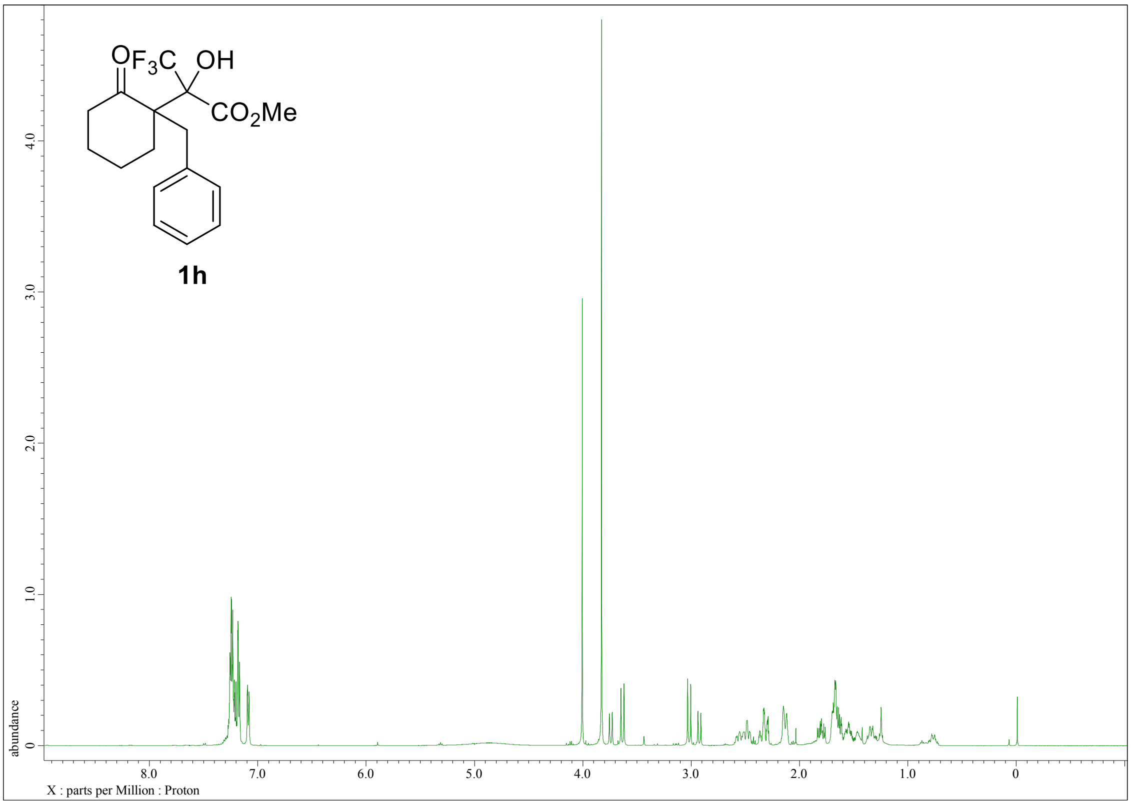

^{1}H NMR spectrum

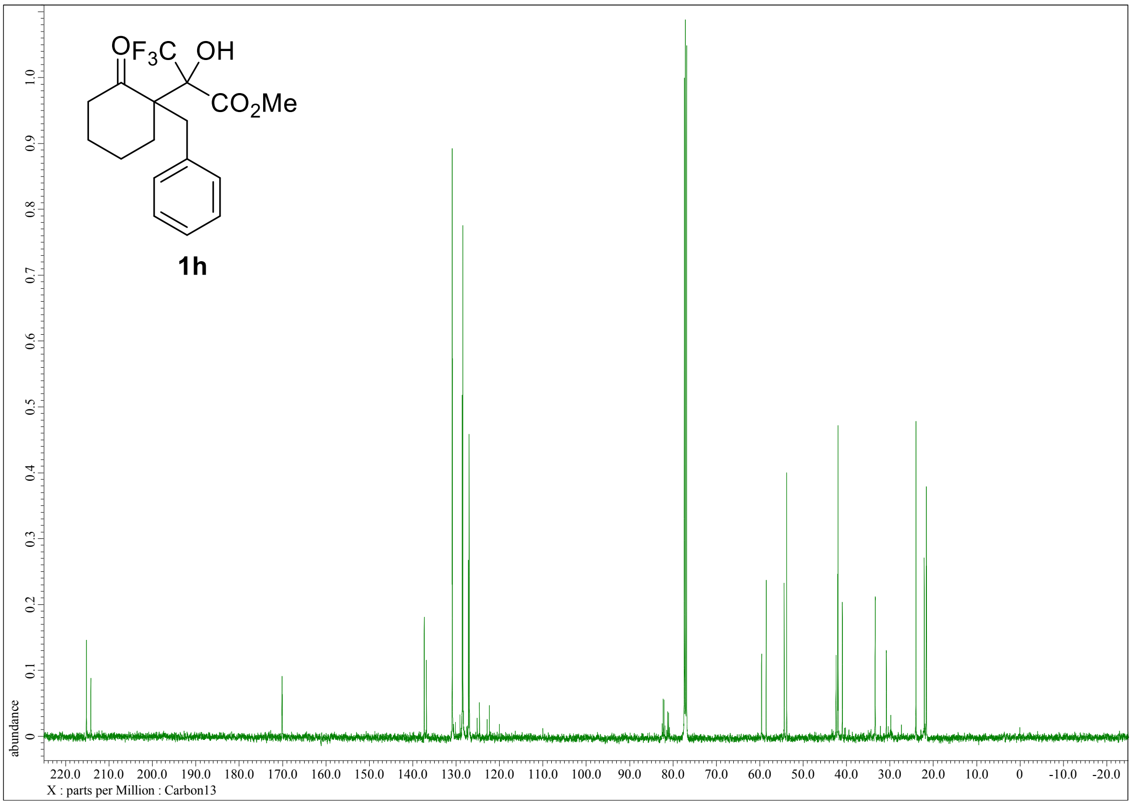

^{13}C NMR spectrum

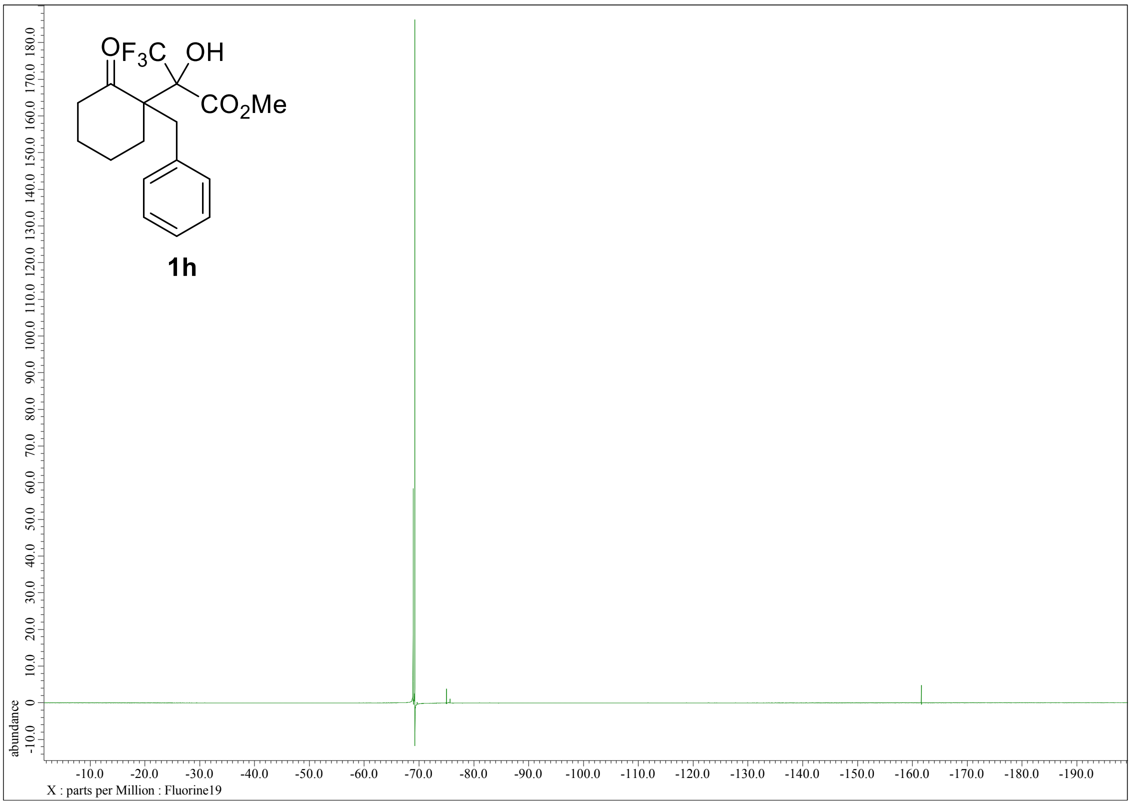

^{19}F NMR spectrum

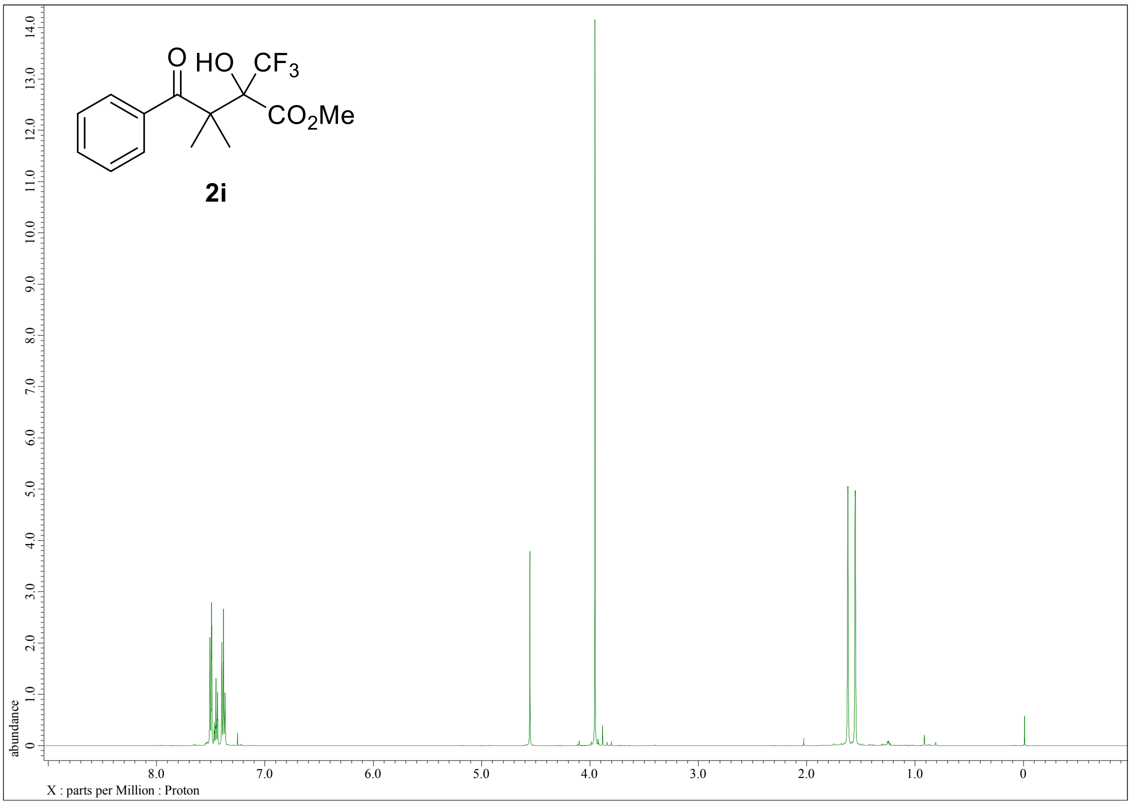

^{1}H NMR spectrum

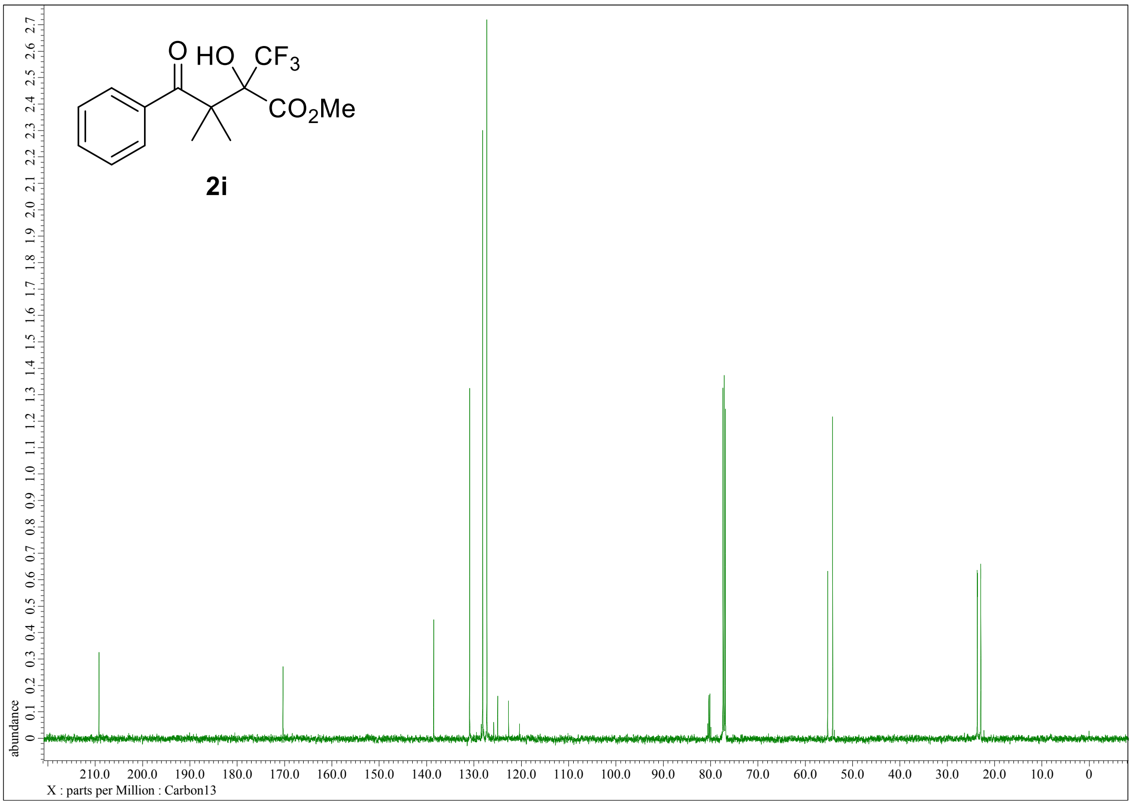

^{13}C NMR spectrum

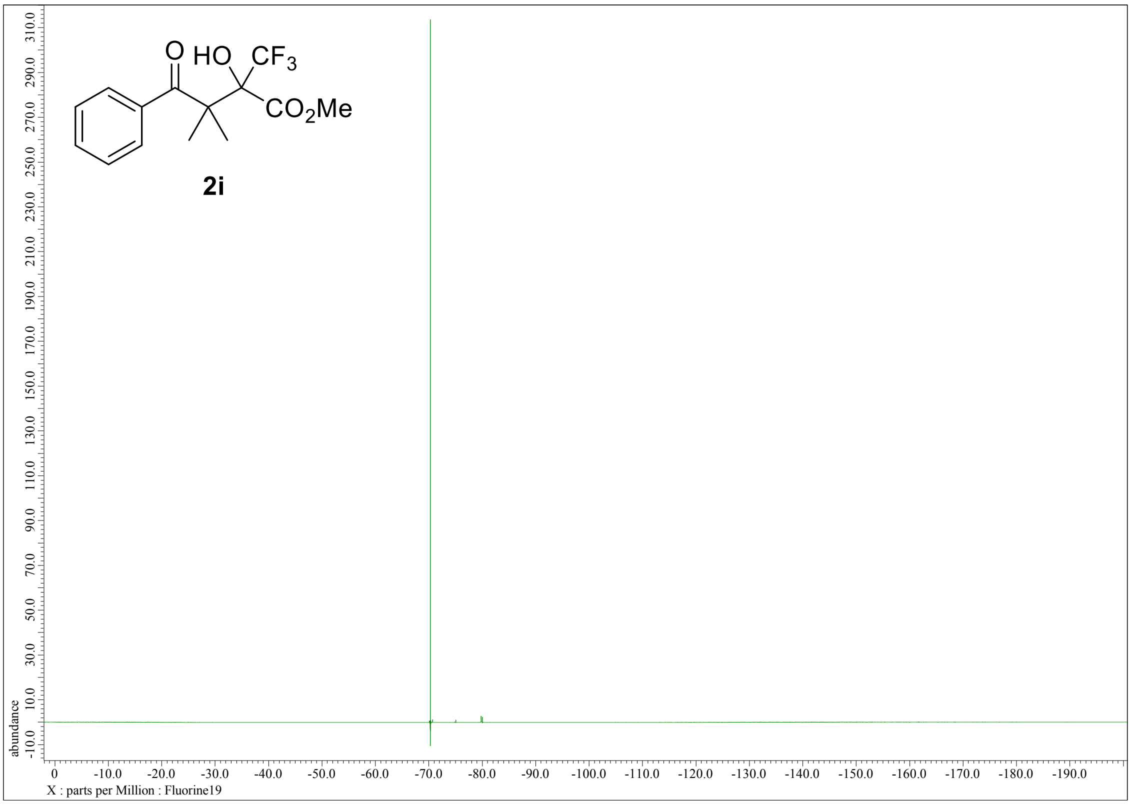

^{19}F NMR spectrum

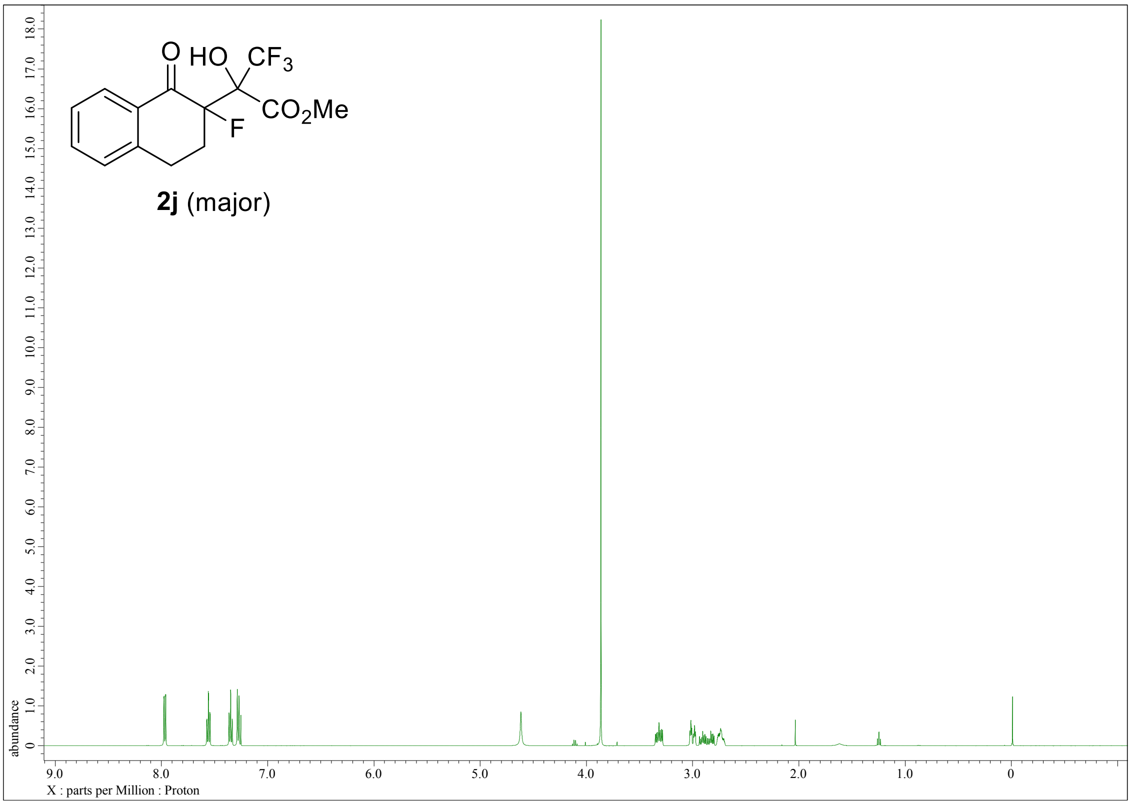

^{1}H NMR spectrum

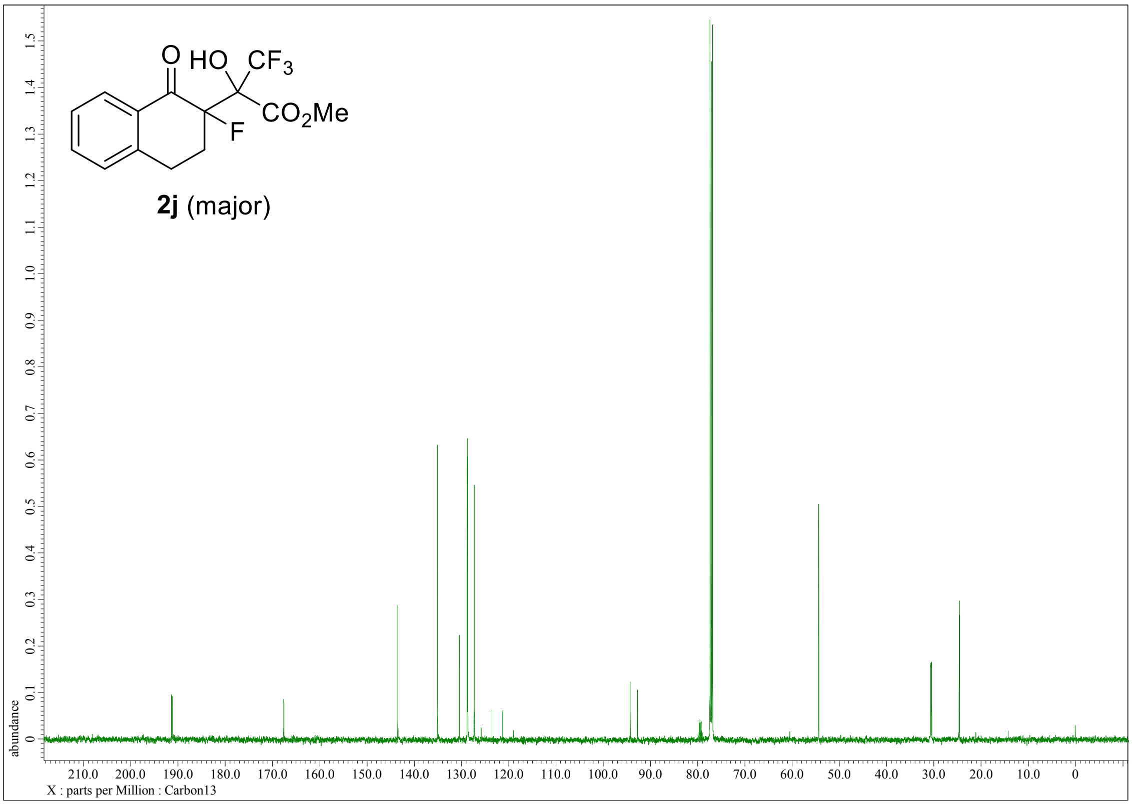

^{13}C NMR spectrum

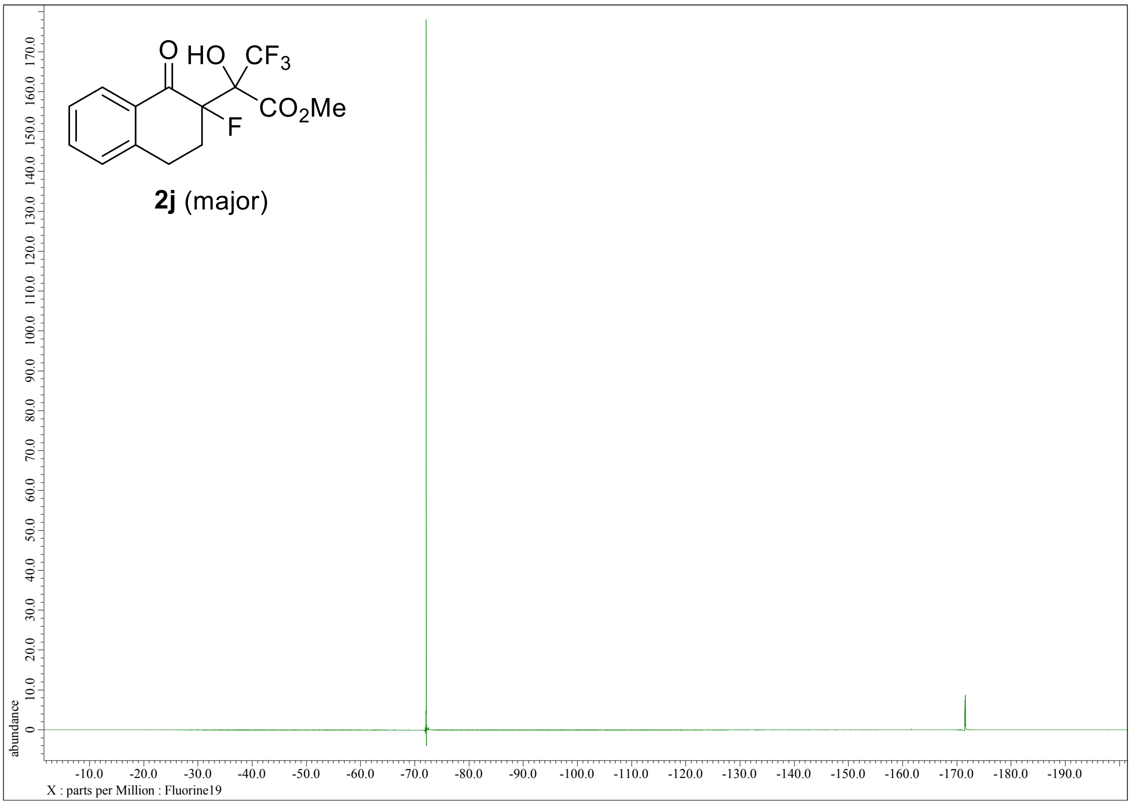

^{19}F NMR spectrum

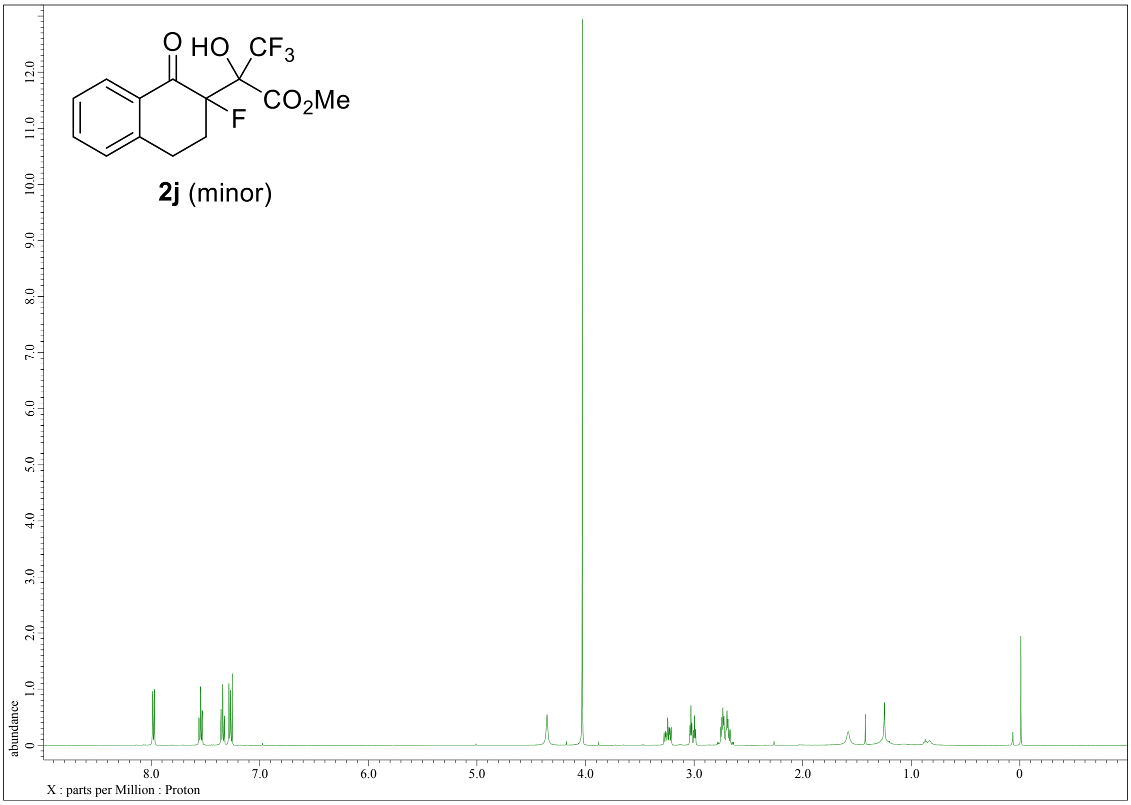

^{1}H NMR spectrum

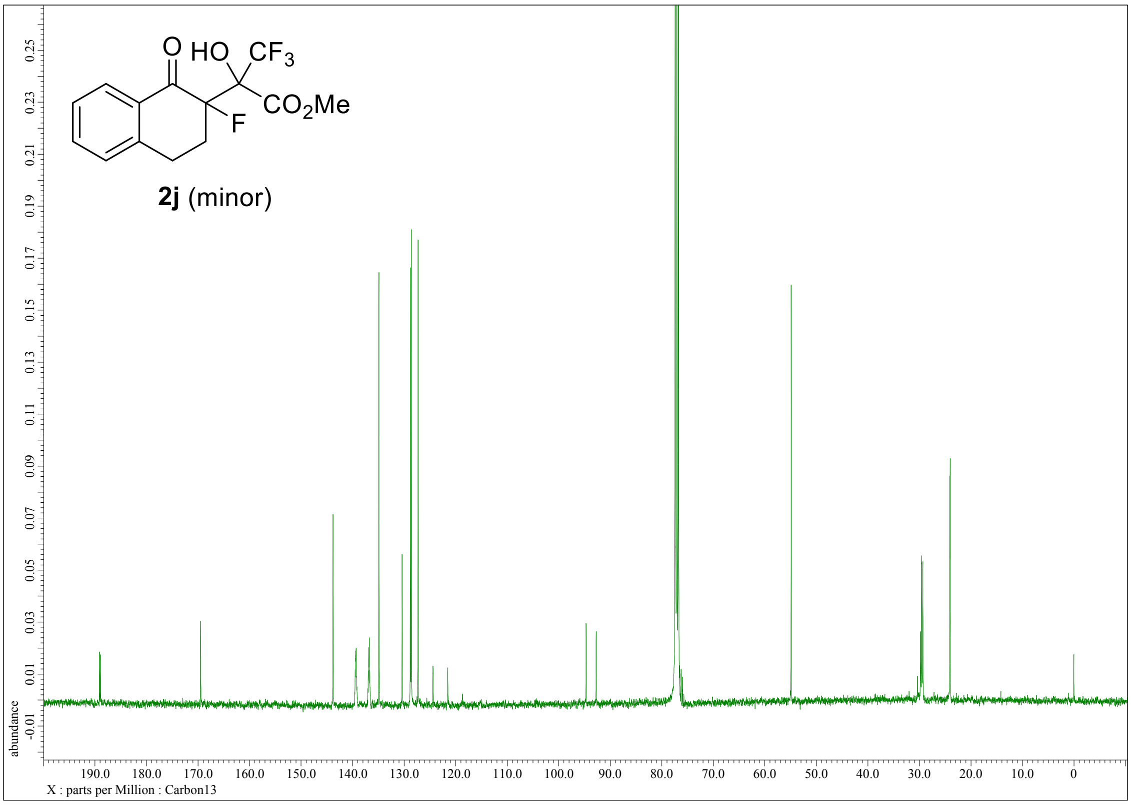

^{13}C NMR spectrum

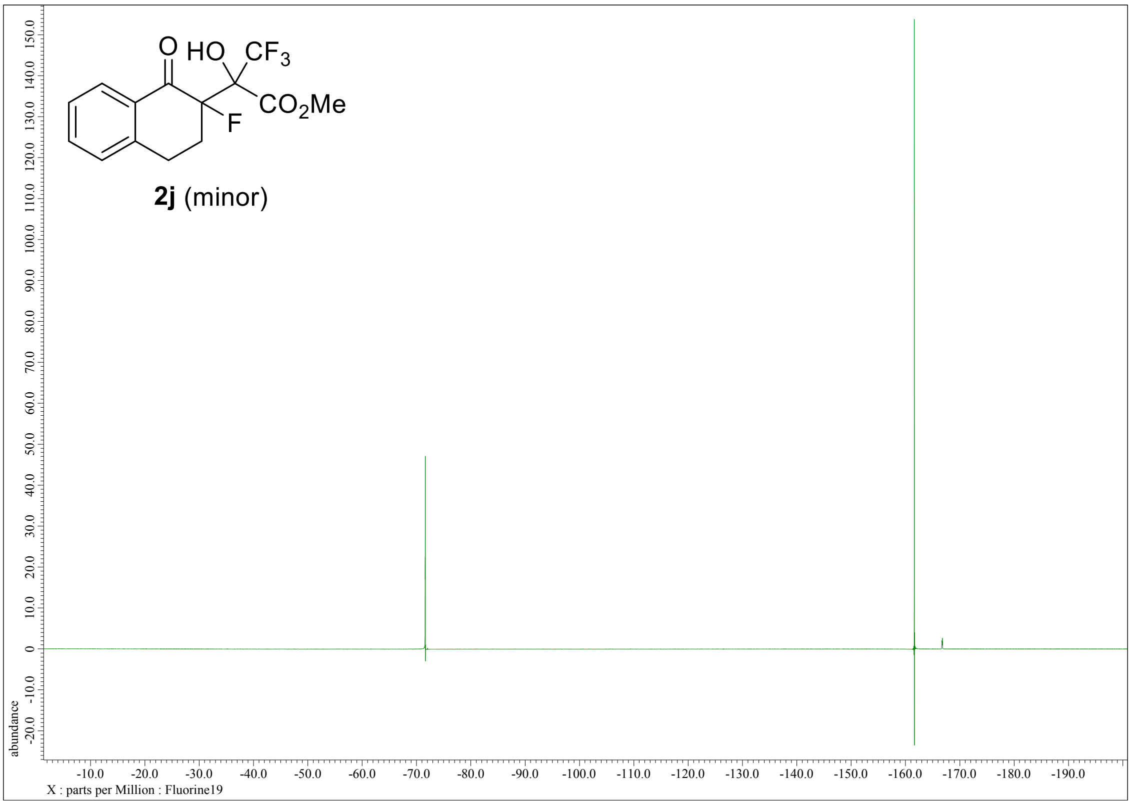

^{19}F NMR spectrum

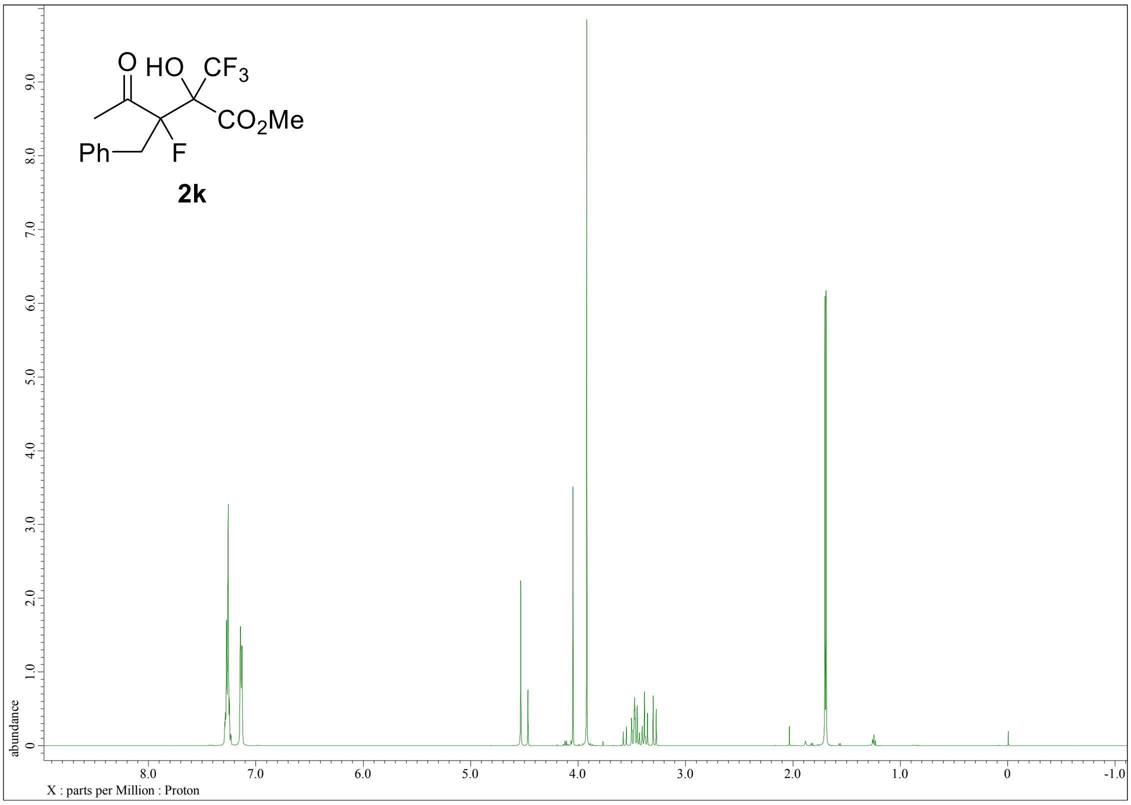

^{1}H NMR spectrum

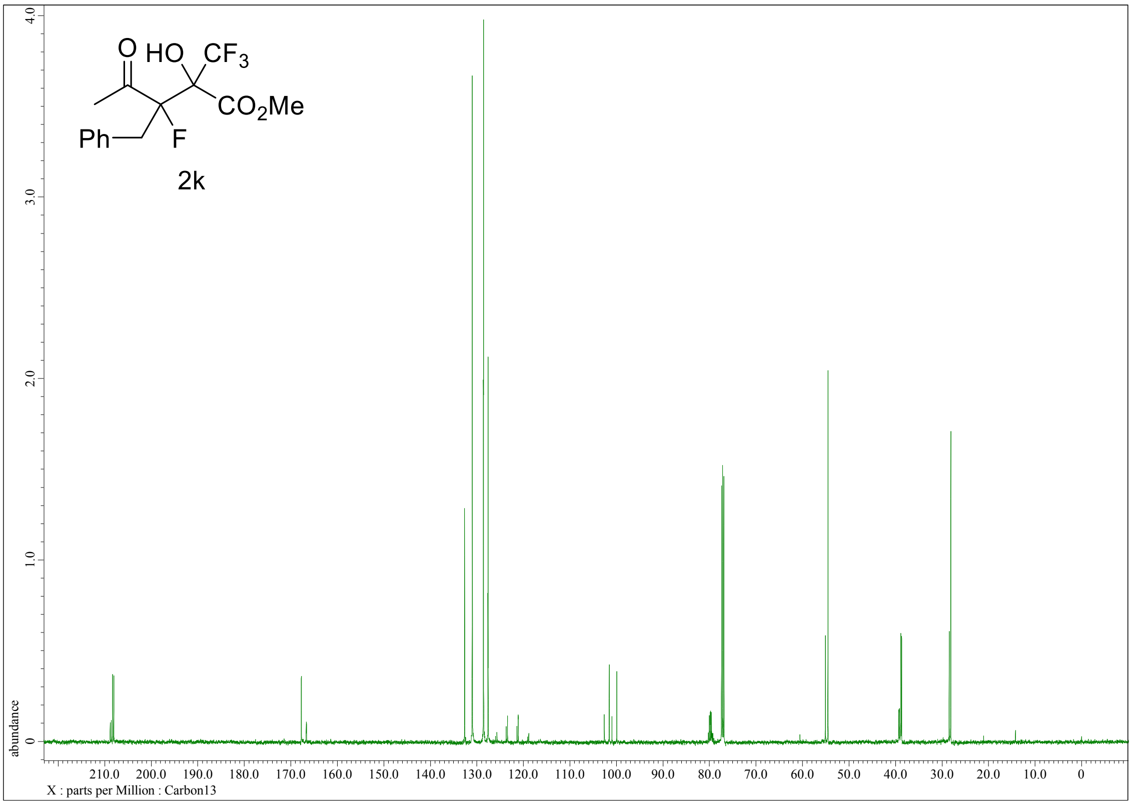

^{13}C NMR spectrum

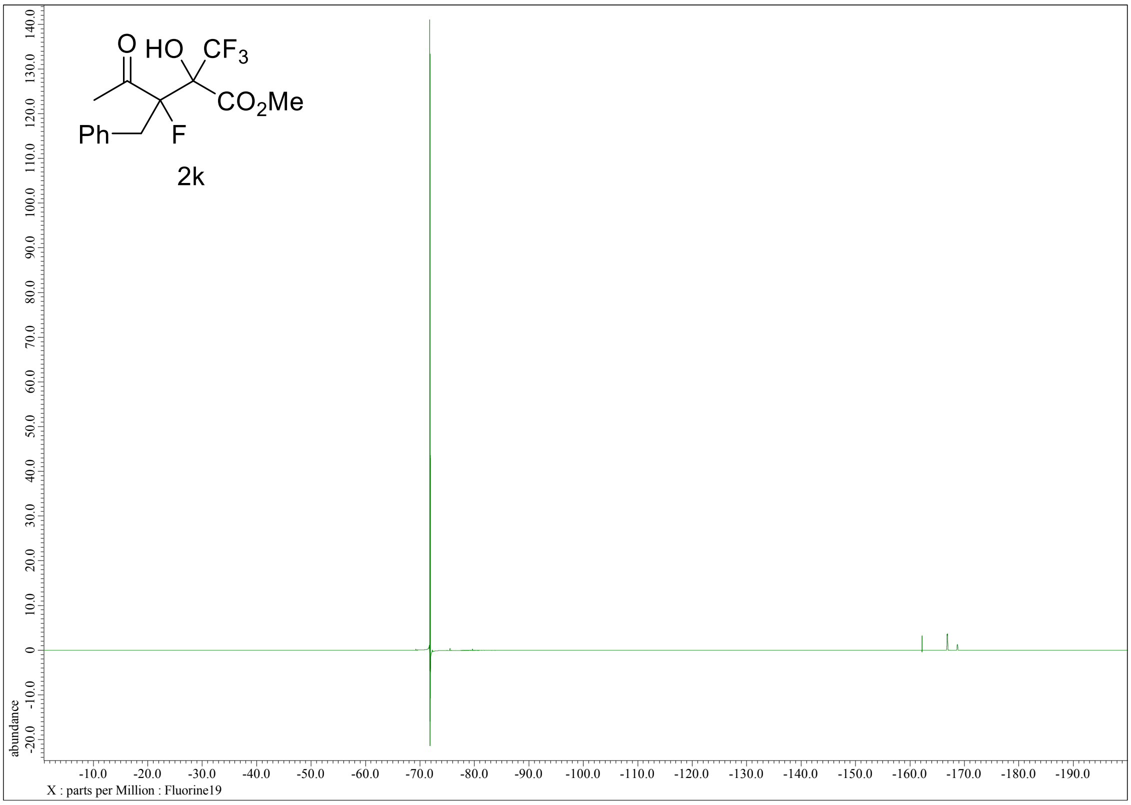

^{19}F NMR spectrum

*Methyl 3,3,3-trifluoro-2-hydroxy-2-(2-methyl-1-oxo-1,2,3,4-tetrahydronaphthalen-2-yl)propanoate(**2a**)*

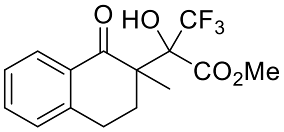

2a

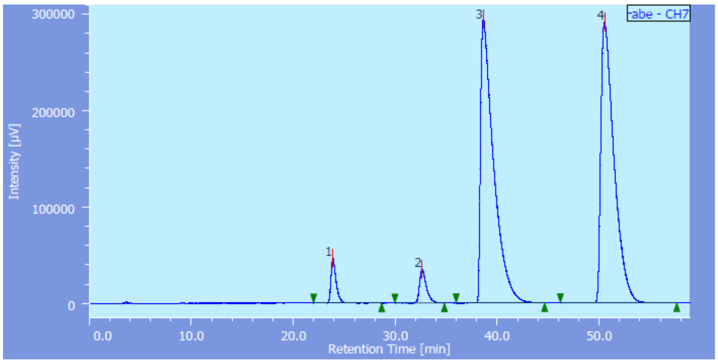

#	ピーク名	CH	tR [min]	面積 [μV·sec]	高さ [μV]	面積%	高さ%	定量値	NTP	分離度	シンメトリー係数	警告
1	Unknown	7	23.865	1613336	45853	2.915	6.890	N/A	11926	8.589	1.609	
2	Unknown	7	32.615	1601466	34479	2.894	5.181	N/A	12458	3.583	1.565	
3	Unknown	7	38.617	26044646	294139	47.058	44.198	N/A	4975	5.302	3.411	
4	Unknown	7	50.510	26085988	291031	47.133	43.731	N/A	7663	N/A	2.327	

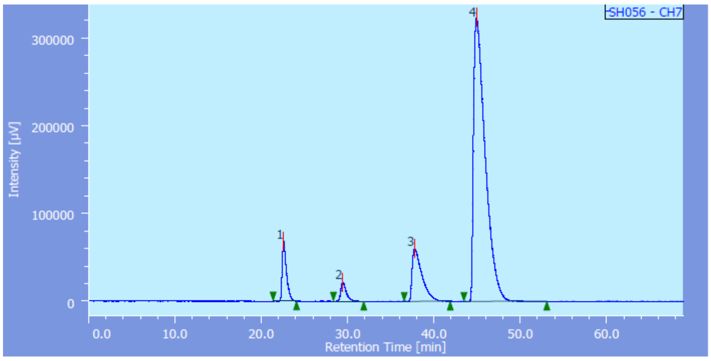

#	ピーク名	CH	tR [min]	面積 [μV·sec]	高さ [μV]	面積%	高さ%	定量値	NTP	分離度	シンメトリー係数	警告
1	Unknown	7	22.588	2303986	67701	6.004	14.320	N/A	11631	7.176	1.796	
2	Unknown	7	29.437	942844	21502	2.457	4.548	N/A	11978	5.417	1.649	
3	Unknown	7	37.752	4735581	59910	12.340	12.672	N/A	5688	3.240	2.653	
4	Unknown	7	44.917	30392152	323665	79.199	68.460	N/A	5456	N/A	2.669	

*Ethyl 3,3,3-trifluoro-2-hydroxy-2-(2-methyl-1-oxo-1,2,3,4-tetrahydronaphthalen-2-yl)propanoate(**2b**)*

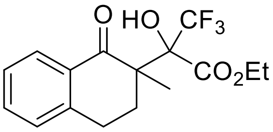

2b

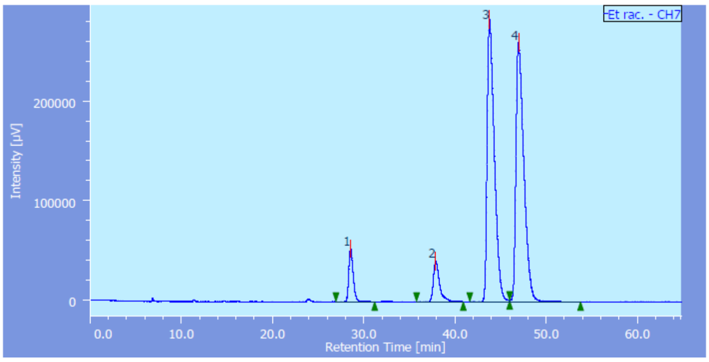

#	ピーク名	CH	tR [min]	面積 [µV·sec]	高さ [µV]	面積%	高さ%	定量値	NTP	分離度	シンメトリー係数	警告
1	Unknown	7	28.553	1775752	53062	5.081	8.330	N/A	17788	9.451	1.341	
2	Unknown	7	37.892	1911489	40924	5.469	6.425	N/A	18150	4.590	1.661	
3	Unknown	7	43.768	15561160	282925	44.521	44.416	N/A	14732	2.120	1.747	
4	Unknown	7	46.955	15703780	260081	44.929	40.830	N/A	14278	N/A	1.732	

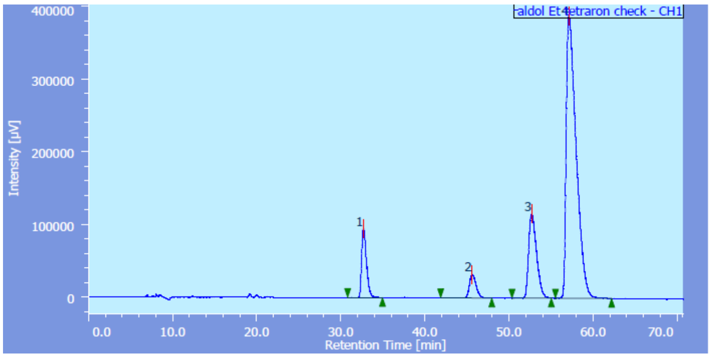

#	ピーク名	CH	tR [min]	面積 [µV·sec]	高さ [µV]	面積%	高さ%	定量値	NTP	分離度	シンメトリー係数	警告
1	Unknown	1	32.692	3782644	94679	8.417	15.022	N/A	15932	10.401	1.477	
2	Unknown	1	45.617	1820468	32537	4.051	5.163	N/A	15706	4.442	1.300	
3	Unknown	1	52.683	7550212	115571	16.801	18.337	N/A	14760	2.289	1.472	
4	Unknown	1	57.125	31784648	387470	70.730	61.478	N/A	11221	N/A	2.177	

*Methyl 2-(7-bromo-2-methyl-1-oxo-1,2,3,4-tetrahydronaphthalen-2-yl)-3,3,3-trifluoro-2-hydroxy propanoate (**2c**)*

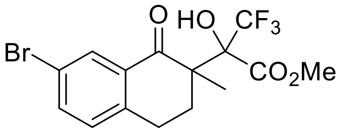

2c

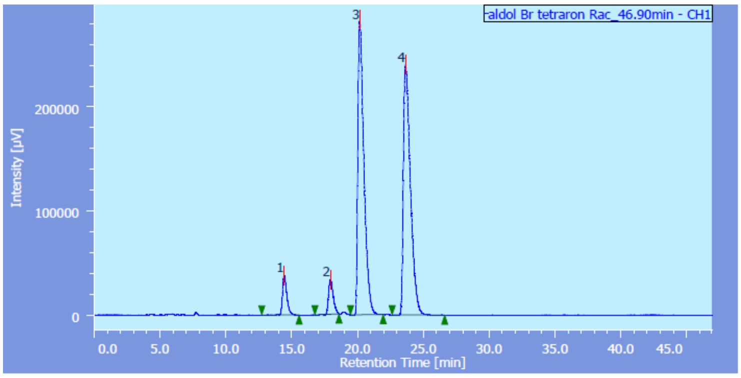

#	ピーク名	CH	tR [min]	面積 [µV·sec]	高さ [µV]	面積%	高さ%	定量値	NTP	分離度	シンメトリー係数	警告
1	Unknown	1	14.433	868243	38205	4.418	6.424	N/A	9933	5.735	1.477	
2	Unknown	1	17.942	822787	33390	4.187	5.614	N/A	12289	3.058	1.402	
3	Unknown	1	20.175	8993823	283042	45.764	47.590	N/A	9756	3.893	1.987	
4	Unknown	1	23.650	8967561	240113	45.631	40.372	N/A	9451	N/A	1.936	

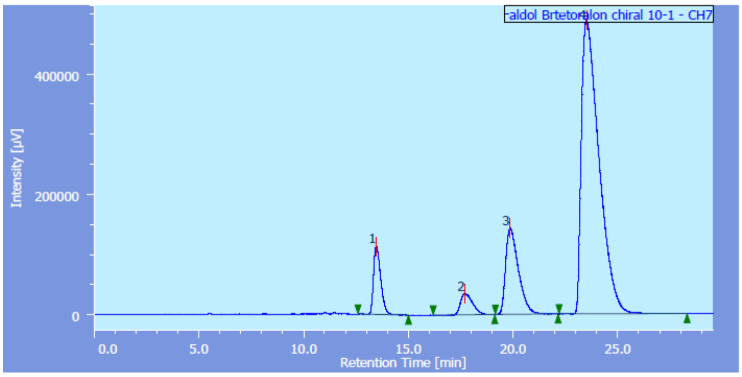

#	ピーク名	CH	tR [min]	面積 [µV·sec]	高さ [µV]	面積%	高さ%	定量値	NTP	分離度	シンメトリー係数	警告
1	Unknown	7	13.467	2709921	113055	6.992	14.512	N/A	7344	4.997	1.488	
2	Unknown	7	17.708	1406092	35093	3.628	4.505	N/A	4353	1.990	1.486	
3	Unknown	7	19.860	6063478	143413	15.645	18.409	N/A	5265	2.772	1.639	
4	Unknown	7	23.507	28576151	487493	73.734	62.575	N/A	3710	N/A	2.173	

*Methyl 2-(2-allyl-1-oxo-1,2,3,4-tetrahydronaphthalen-2-yl)-3,3,3-trifluoro-2-hydroxypropanoate (**2d**)*

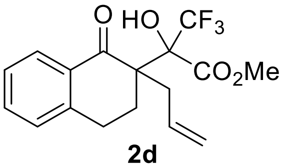

2d

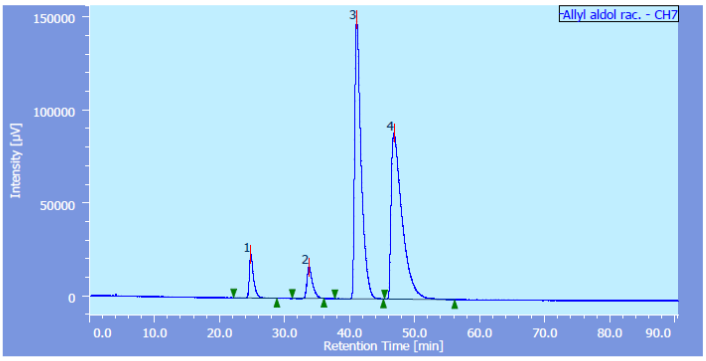

#	ピーク名	CH	tR [min]	面積 [µV·sec]	高さ [µV]	面積%	高さ%	定量値	NTP	分離度	シンメトリー係数	警告
1	Unknown	7	24.825	1041885	23285	4.535	8.342	N/A	8349	7.026	1.654	
2	Unknown	7	33.772	992769	16872	4.321	6.044	N/A	8483	4.584	1.558	
3	Unknown	7	41.090	10477379	149879	45.606	53.695	N/A	8977	2.480	2.050	
4	Unknown	7	46.827	10461792	89095	45.538	31.919	N/A	4158	N/A	2.722	

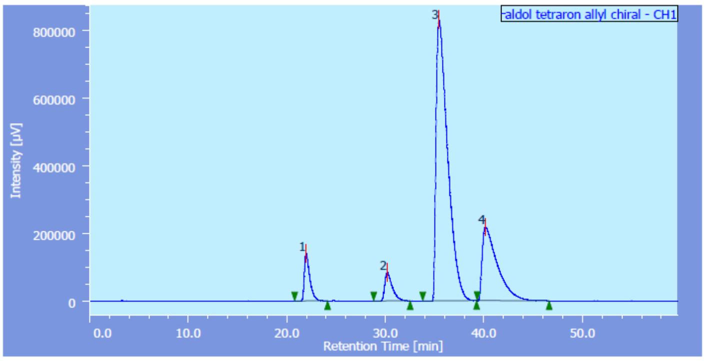

#	ピーク名	CH	tR [min]	面積 [µV·sec]	高さ [µV]	面積%	高さ%	定量値	NTP	分離度	シンメトリー係数	警告
1	Unknown	1	21.967	5325797	139534	5.290	10.990	N/A	8556	7.203	1.837	
2	Unknown	1	30.183	4412723	83145	4.383	6.549	N/A	8145	3.043	1.866	
3	Unknown	1	35.450	67631832	829473	67.179	65.334	N/A	4422	1.971	2.548	
4	Unknown	1	40.158	23303011	217443	23.147	17.127	N/A	3655	N/A	3.213	

Methyl 3,3,3-trifluoro-2-hydroxy-2-(2-methyl-1-oxo-2,3-dihydro-1H-inden-2-yl)propanoate (***2e***)

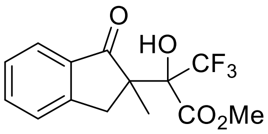

2e

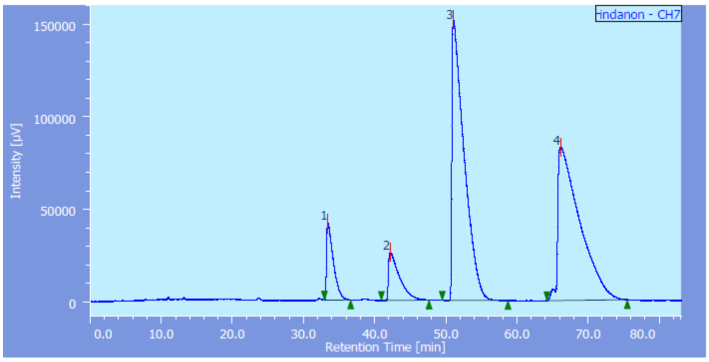

#	ピーク名	CH	tR [min]	面積 [µV·sec]	高さ [µV]	面積%	高さ%	定量値	NTP	分離度	シンメトリー係数	警告
1	Unknown	7	33.415	2767059	41735	6.686	13.808	N/A	6254	4.098	3.469	
2	Unknown	7	42.183	2765058	26047	6.681	8.617	N/A	4211	3.150	4.690	
3	Unknown	7	51.068	18010482	151582	43.516	50.150	N/A	4465	3.566	5.479	
4	Unknown	7	66.135	17845716	82895	43.118	27.425	N/A	2386	N/A	2.976	

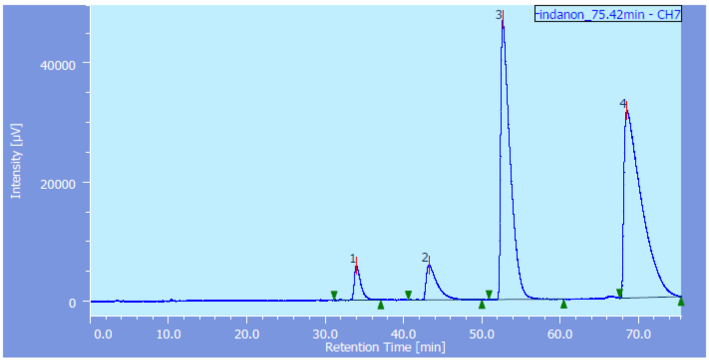

#	ピーク名	CH	tR [min]	面積 [µV·sec]	高さ [µV]	面積%	高さ%	定量値	NTP	分離度	シンメトリー係数	警告
1	Unknown	7	34.017	334759	5778	3.434	6.417	N/A	9077	4.881	1.831	
2	Unknown	7	43.238	572857	5894	5.876	6.545	N/A	5368	4.210	2.992	
3	Unknown	7	52.660	3925206	46929	40.264	52.114	N/A	9809	5.192	3.008	
4	Unknown	7	68.500	4915802	31449	50.426	34.924	N/A	4713	N/A	4.363	

*Methyl 3,3,3-trifluoro-2-hydroxy-2-(6-methyl-5-oxo-6,7,8,9-tetrahydro-5H-benzo[7]annulen-6-yl)propanoate (**2g**)*

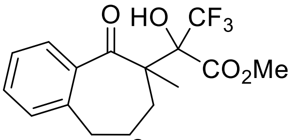

2g

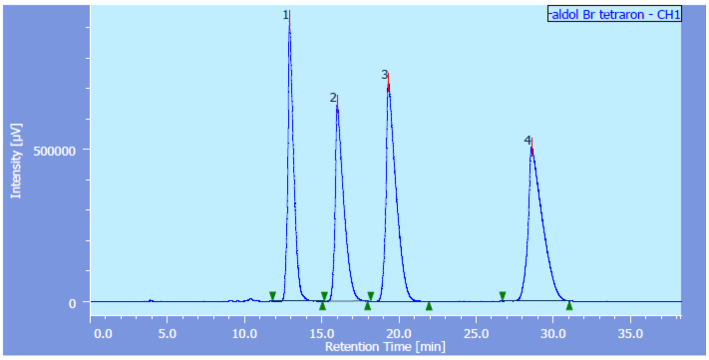

#	ピーク名	CH	tR [min]	面積 [μV·sec]	高さ [μV]	面積%	高さ%	定量値	NTP	分離度	シンメトリー係数	警告
1	Unknown	1	12.917	24151750	923434	21.222	33.040	N/A	6953	3.982	1.568	
2	Unknown	1	16.000	24700247	645778	21.704	23.106	N/A	4704	3.257	2.024	
3	Unknown	1	19.317	32325360	720097	28.404	25.765	N/A	4855	6.817	1.881	
4	Unknown	1	28.583	32629317	505574	28.671	18.089	N/A	4998	N/A	1.804	

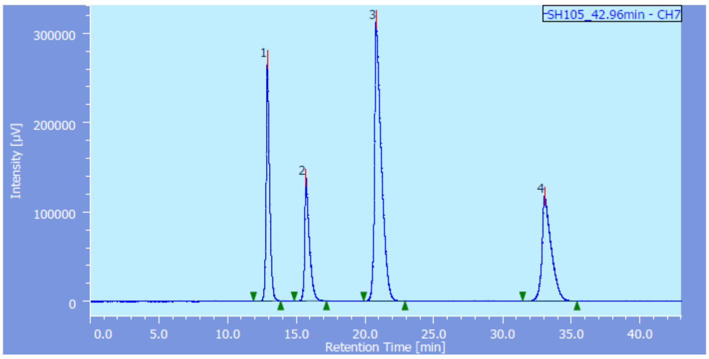

#	ピーク名	CH	tR [min]	面積 [μV·sec]	高さ [μV]	面積%	高さ%	定量値	NTP	分離度	シンメトリー係数	警告
1	Unknown	7	12.895	4525778	269835	18.913	32.131	N/A	16520	5.872	1.412	
2	Unknown	7	15.700	3242107	137898	13.549	16.421	N/A	12732	7.354	1.800	
3	Unknown	7	20.800	10691183	314643	44.679	37.467	N/A	9950	12.483	1.934	
4	Unknown	7	33.040	5469774	117417	22.858	13.982	N/A	13626	N/A	1.631	

*Methyl 2-hydroxy-3,3-dimethyl-4-oxo-4-phenyl-2-(trifluoromethyl)butanoate (**2i**)*

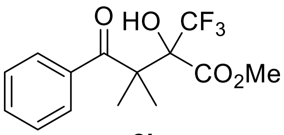

2i

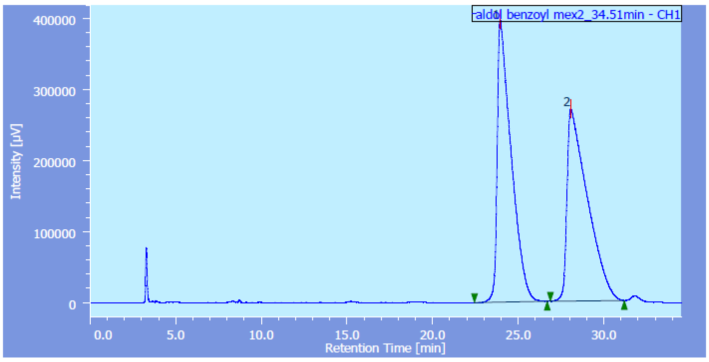

#	ピーク名	CH	tR [min]	面積 [µV·sec]	高さ [µV]	面積%	高さ%	定量値	NTP	分離度	シンメトリー係数	警告
1	Unknown	1	23.942	22495812	397173	49.986	59.460	N/A	4305	2.259	2.451	
2	Unknown	1	28.058	22508208	270792	50.014	40.540	N/A	2613	N/A	2.924	

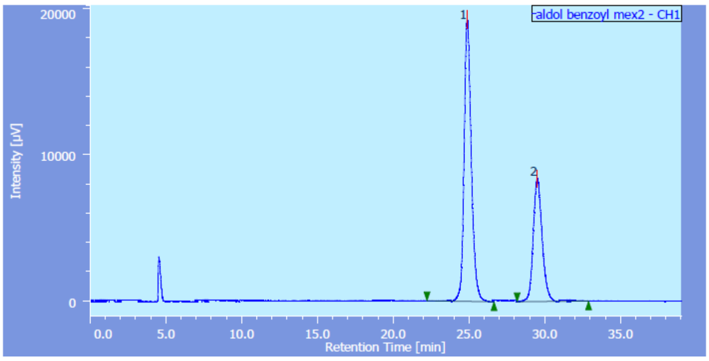

#	ピーク名	CH	tR [min]	面積 [µV·sec]	高さ [µV]	面積%	高さ%	定量値	NTP	分離度	シンメトリー係数	警告
1	Unknown	1	24.883	635968	19268	64.715	69.626	N/A	14469	4.962	1.225	
2	Unknown	1	29.508	346753	8405	35.285	30.374	N/A	12844	N/A	1.164	

Methyl 3,3,3-trifluoro-2-(2-fluoro-1-oxo-1,2,3,4-tetrahydronaphthalen-2-yl)-2hydroxypropanoate (***2j***)

major isomer

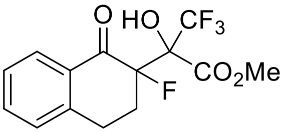

2j

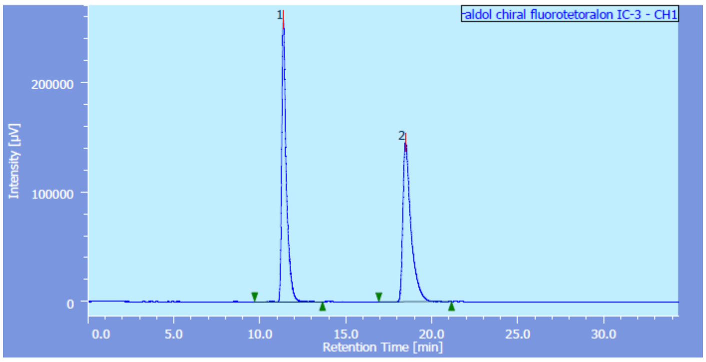

#	ピーク名	CH	tR [min]	面積 [μV·sec]	高さ [μV]	面積%	高さ%	定量値	NTP	分離度	シンメトリー係数	警告
1	Unknown	1	11.367	4769442	257088	50.064	63.883	N/A	11238	11.789	1.791	
2	Unknown	1	18.475	4757154	145348	49.936	36.117	N/A	8972	N/A	1.968	

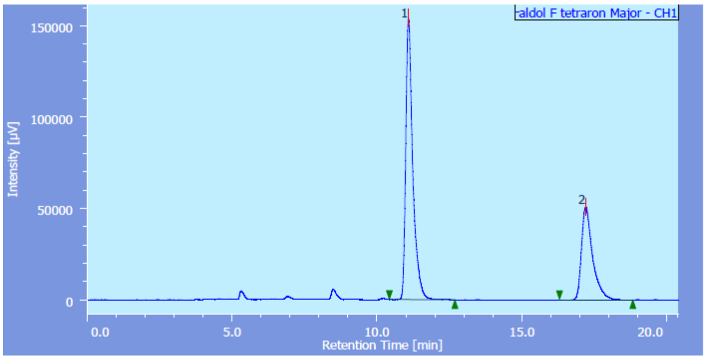

#	ピーク名	CH	tR [min]	面積 [μV·sec]	高さ [μV]	面積%	高さ%	定量値	NTP	分離度	シンメトリー係数	警告
1	Unknown	1	11.083	2587457	153850	65.889	75.196	N/A	12143	11.756	1.615	
2	Unknown	1	17.200	1339541	50748	34.111	24.804	N/A	11519	N/A	1.613	

Methyl 3,3,3-trifluoro-2-(2-fluoro-1-oxo-1,2,3,4-tetrahydronaphthalen-2-yl)-2-hydroxypropanoate (***2j***)
minor isomer

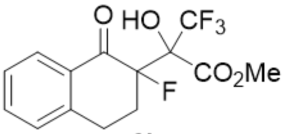

2j

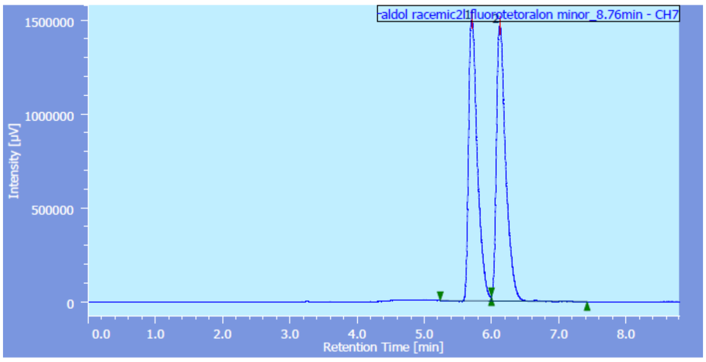

#	ピーク名	CH	tR [min]	面積 [μV·sec]	高さ [μV]	面積%	高さ%	定量値	NTP	分離度	シンメトリー係数	警告
1	Unknown	7	5.705	13657376	1495117	49.097	50.501	N/A	9943	1.779	1.677	
2	Unknown	7	6.120	14159882	1465439	50.903	49.499	N/A	10495	N/A	1.688	

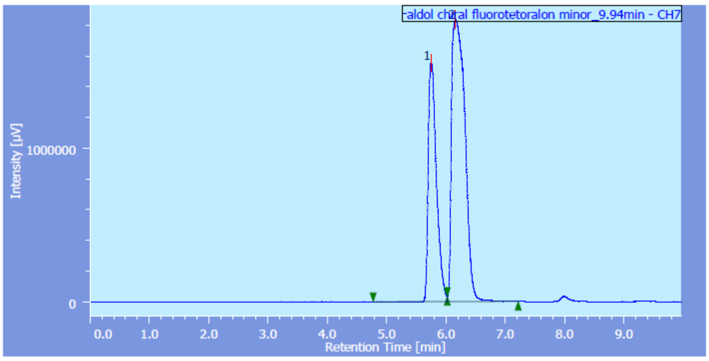

#	ピーク名	CH	tR [min]	面積 [μV·sec]	高さ [μV]	面積%	高さ%	定量値	NTP	分離度	シンメトリー係数	警告
1	Unknown	7	5.748	14995014	1554230	34.352	45.872	N/A	8748	1.215	1.716	
2	Unknown	7	6.163	28655448	1833928	65.648	54.128	N/A	3149	N/A	1.771	
